# Unveiling the anti-cancer mechanism for half-sandwich and cyclometalated Ir(iii)-based complexes with functionalized α-lipoic acid†

**DOI:** 10.1039/c9ra10357k

**Published:** 2020-02-03

**Authors:** Meng-Meng Wang, Xu-Ling Xue, Xi-Xi Sheng, Yan Su, Ya-Qiong Kong, Yong Qian, Jian-Chun Bao, Zhi Su, Hong-Ke Liu

**Affiliations:** Jiangsu Collaborative Innovation Center of Biomedical Functional Materials, College of Chemistry and Materials Science, Nanjing Normal University Nanjing 210023 China zhisu@njnu.edu.cn liuhongke@njnu.edu.cn

## Abstract

Alpha lipoic acid (LA) is a natural compound and coenzyme with sufficient safety information for serving as a promising anticancer agent. To further clarify the mechanism of action (MoA), two Ir(iii) complexes with the functionalized α-lipoic acid (N^∧^N-LA, N^∧^N, 2,2-bipyridine derivative), namely Ir1 and Ir2, were synthesized, where Ir1 possessed a half-sandwich structure with the formula [Ir(Cp*)(N^∧^N-LA)Cl]PF_6_ (Cp* = 1,2,3,4,5-pentamethyl-cyclopentadiene) and Ir2 possessed the cyclometalated structure with the formula [Ir(C^∧^N)_2_(N^∧^N-LA)]PF_6_ (C^∧^N = 2-phenylpyridine). Even though both complexes were constructed based on the same N^∧^N-LA ligand, Ir1 showed no cytotoxicity (IC_50_ > 200 μM), which was due to its low lipophilicity for hard penetration into the cancer cells, easy hydrolysis, and reaction with GSH. Ir2 exhibited excellent cytotoxicity (IC_50_ = 3.43–6.74 μM) toward diverse cancer cell lines *in vitro* and a promising ability to overcome the cisplatin-resistance in A549R cells. The anticancer mechanism of Ir2 in A549 cells was investigated in detail, and it was found it could localize and accumulate in the lysosomes of A549 cells, induce ROS, arrest the cycle at G_0_/G_1_, and lead to cell death by autophagy. Comparison with Ir-NH_2_ ([Ir(C^∧^N)_2_(N^∧^N-NH_2_)]PF_6_) demonstrated that introduction of the LA ligand to Ir2 could highly enhance the cytotoxicity and help to overcome the cisplatin-resistance. This study of the half-sandwich and cyclometalated Ir(iii)-based anticancer agents highlighted the different MoAs toward cancer cells and provided new insights for understanding their structure–property relationships.

## Abstract 2

The introduction of LA improved the anticancer activity of the complex and helped overcome the cisplatin-resistance.

## Graphical abstract

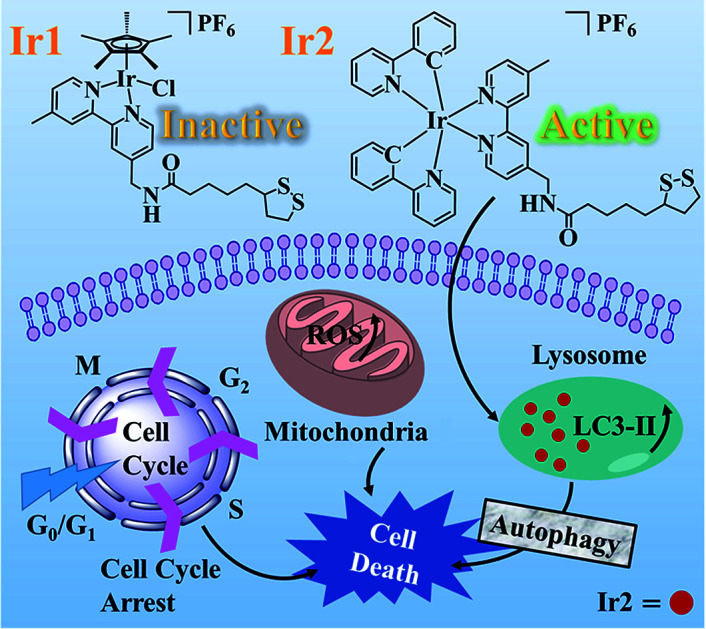

## Introduction

The endogenous disulfide α-lipoic acid (LA) is accepted as a promising anticancer agent due to its potent activities toward various cancer cell lines at concentrations in the mM range.^1,2^ Most importantly, LA is a natural compound and coenzyme with sufficient safety information,^3,4^ which facilitates its use in cancer patients to support chemotherapy. It has been previously reported that LA could initiate the apoptosis of various cancer cells,^5–7^ which is the primary mechanism of anticancer-drugs-induced cell death. Even the role of autophagy (an alternative cell death pathway) in cancer treatment is complicated, and modulating autophagy has recently emerged as a promising therapeutic approach.

As is known, metal-based anticancer agents have exhibited excellent efficiency in clinical treatment toward various types of carcinomas over the past several decades.^8,9^ However, several physiological limits and clinical issues have arisen with cisplatin and its derivatives, such as poor selectivity, serious toxic side effects, strong drug resistance, and low biological utilities.^10,11^ Hence, there is an urgent need to develop new generations of metal anticancer agents in the field of metallodrugs.^12–15^ Recently, the third-row transition metal iridium (Ir) has attracted much attention due to its variety of oxidation states, coordination numbers, coordination geometries, and catalytic properties.^16,17^ Sadler and Liu *et al.* established a clear relationship between cyclopentadienyl ligands and the anticancer activity of half-sandwich Ir(iii) complexes.^18,19^ Chao *et al.* achieved the organelle-targeting of cyclometalated Ir(iii) complexes by regulating the relevant ligands to adjust their lipophilicity.^20^

Herein, the half-sandwich iridium(iii) complex Ir1 containing the modified α-lipoic acid ligand (N^∧^N-LA, N^∧^N, 2,2-bipyridine derivative) was synthesized to try to combine the dual function of LA and the transition metal. However, it was found that the “piano-stool” complex Ir1 was nearly inactive toward the tested cancer cell lines, attributed to the low lipophilicity and low cellular uptake. In contrast, the biological properties were greatly improved by the cyclometalated complex Ir2, which resulted from the N^∧^N-LA ligand reaction with the dinuclear precursor [Ir(ppy)_2_Cl]_2_ (ppy = 2-phenylpyridine). To demonstrate the importance of the LA ligand, Ir-NH_2_ was used as a model complex of Ir2 in this work. Ir2 exhibited enhanced cytotoxicity to the cancer cells at concentrations at the μM level and showed a promising ability to overcome the cisplatin-resistance, which demonstrated that the anticancer activities of the metallodrugs were highly dependent on their affiliated structures.^21,22^

## Results and discussion

### Syntheses and characterization

**Scheme 1:**
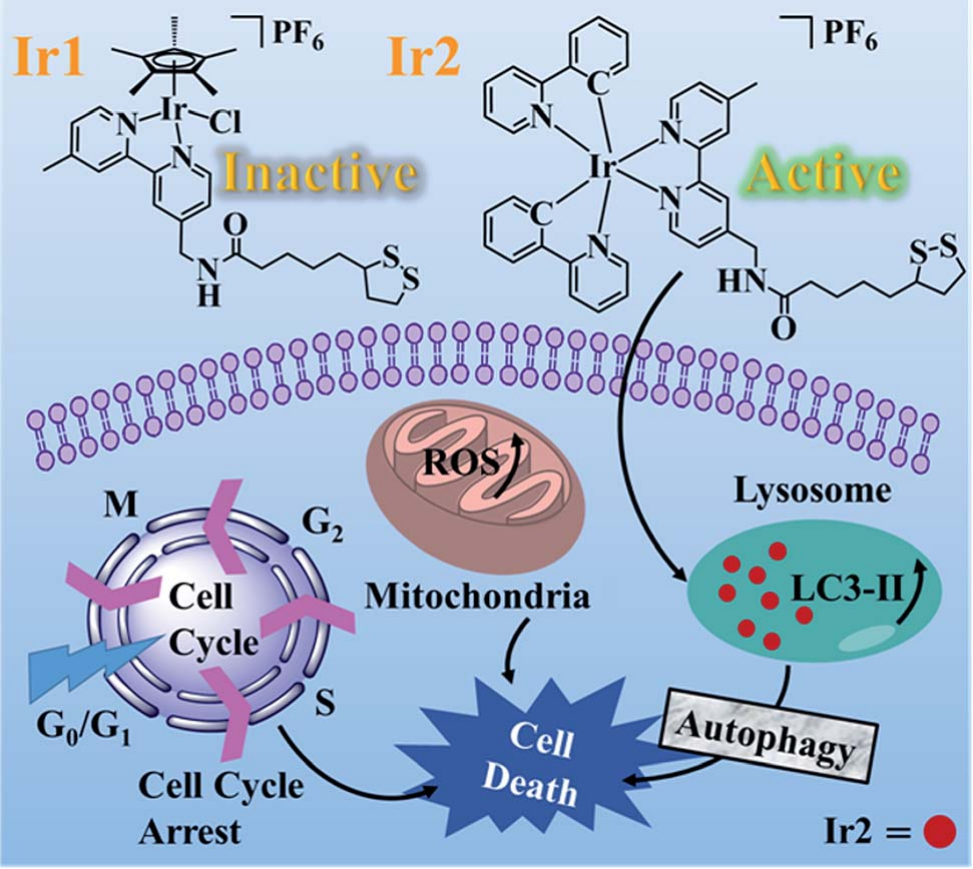
Chemical structures and distinct anticancer mechanism of complexes Ir1 and Ir2.

Complexes Ir1 and Ir2 were synthesized from the functionalized α-lipoic acid ligand (N^∧^N-LA) with distinct dinuclear precursors [(η^5^-Cp*)IrCl_2_]_2_ and [Ir(ppy)_2_Cl]_2_, respectively (Scheme S1†). Complex Ir1 was a typical half-sandwich “piano-stool” Ir(iii) complex with the formula [Ir(Cp*)(N^∧^N-LA)Cl]PF_6_ (Cp* = 1,2,3,4,5-pentamethyl-cyclopentadiene), while complex Ir2 was a cyclometalated Ir(iii) complex with the formula [Ir(C^∧^N)_2_(N^∧^N-LA)]PF_6_ (C^∧^N = 2-phenylpyridine) (Scheme 1). The model complex of Ir2, named Ir-NH_2_, was synthesized to evaluate the biological function of LA, where the N^∧^N-LA ligand was replaced by the 4-aminomethyl-4-methyl-2,2-bipyridyl. Pure products of all these three complexes were recrystallized from CH_2_Cl_2_/CH_3_OCH_3_ and were fully characterized by ^1^H NMR, ^13^C NMR, and ESI-MS (Fig. S1–S11†).

### Stability and photophysical properties

To confirm the structural stability of Ir1 and Ir2 in aqueous solution, UV-vis spectra of both complexes in DMSO/H_2_O (v/v, 5 : 95) were recorded over 24 h at 298 K (Fig. S12 and S13†). In the UV-vis spectra (Fig. S12a†), the relatively low-energy bands around 310 nm for both Ir1 and Ir2 resulted from the mixed singlet and triplet metal-to-ligand charge-transfer (^1^MLCT and ^3^MLCT) and ligand-to-ligand charge-transfer (LLCT) transitions.^23,24^ The intense high-energy band at 255 nm for Ir2 could be assigned to spin-allowed ligand-centered (^1^LC) π–π* transitions for the cyclometalated (C^∧^N) and ancillary (N^∧^N) ligands.^25^

According to the literature, the metal-based half-sandwich complexes were easy to hydrolyze in the aqua solution, due to the presence of solvent molecules.^26^ Complex Ir1 showed a similar behavior, where the absorbance at 314 nm gradually decreased over 24 h in the UV-vis spectrum (Fig. S13a†). The hydrolysis rate constant and half-life for Ir1 were also determined (Table S1†), which were longer than reported for the Ir piano stool complex,^27^ 0.093 h^−1^*vs.* 0.0084 min^−1^ and 7.5 h *vs.* 82.2 min, respectively.

The absorbance of complex Ir2, however, showed nearly no obvious change under the same conditions (Fig. S13b†), suggesting the cyclometalated complex Ir2 was stable in the aqua solution. The stability of complexes Ir1 and Ir2 (10 mM) were further confirmed by ^1^H NMR recorded over a period of 48 h in *d*_6_-DMSO/D_2_O (v/v, 3 : 1) at 298 K (Fig. S14†). After 8 h, new peaks in the aromatic region appeared in the ^1^H NMR spectrum of Ir1, which arose from the partial hydrolysis of complex Ir1.^18,19^ As expected, the spectrum of Ir2 showed no change over a 48 h period, confirming the high stability of Ir2.

In addition, the photoluminescence spectra of both complexes Ir1 and Ir2 were also investigated under the excitation at 405 nm. Half-sandwich Ir1 showed no emission in the range of 500–800 nm, which is consistent with the previous reports.^28^ Nevertheless, the cyclometalated complex Ir2 exhibited strong red fluorescence at 605 nm (Fig. S12b†), which could be utilized to locate Ir2 in the intracellular biological experiments.

### Evaluation of cytotoxicity

The anti-proliferative activities of Ir1, Ir-NH_2_, and Ir2 were determined against human lung carcinoma (A549), cisplatin-resistant A549 (A549R), human breast carcinoma (MCF-7), epithelial ovarian carcinoma (A2780), human cervical carcinoma (HeLa), and human normal liver (LO2) cells by 3-(4,5-dimethylthiazol-2-yl)-2,5-diphenyltetrazolium bromide (MTT) assay after 48 h treatment (Table 1). The α-lipoic acid (LA) and the functionalized N^∧^N-LA ligand were also evaluated for comparison. LA itself and complex Ir1 were inactive to all the tested cancer cell lines (IC_50_ > 200 μM). Compared to LA itself, the functionalized N^∧^N-LA, however, indicated a higher cytotoxicity to certain cancer cells (such as MCF-7, A2780, and HeLa) with IC_50_ values ranging from 67.5 to 138.6 μM. Complex Ir2 indicated the highest cytotoxicity among the listed complexes with IC_50_ values ranging from 3.4 to 6.7 μM for 48 h, whereby the anticancer activity was even better than that of cis-Pt.

**Table 1:** IC_50_ values of LA, N^∧^N-LA, and complexes synthesized toward different cancer cell lines^a^

Complex	IC_50_ (μM)					
	A549	A549R	MCF-7	A2780	HeLa	LO2
LA	>200	>200	>200	>200	>200	>200
N^∧^N-LA	>200	>200	138.6 ± 2.2	84.5 ± 4.1	67.5 ± 5.0	96.0 ± 6.7
Ir1	>200	>200	>200	>200	>200	>200
Ir-NH_2_	11.2 ± 0.65	19.0 ± 0.59	11.6 ± 0.48	8.08 ± 0.20	21.0 ± 0.95	8.97 ± 0.55
Ir2	6.74 ± 0.41	6.42 ± 0.33	4.93 ± 0.17	3.43 ± 0.12	6.02 ± 0.16	3.84 ± 0.36
cis-Pt	10.5 ± 0.63	17.9 ± 0.77	8.70 ± 0.57	4.32 ± 0.25	7.59 ± 0.46	3.97 ± 0.25

a IC_50_ values are given in μM, and cisplatin (*cis*-Pt) is included for comparison. Data are presented as the mean value ± standard deviation. Cell viability was assessed after 48 h of incubation.

To demonstrate that the introduction of LA into complex Ir2 could enhance the bioactivity, the anti-proliferative activities for Ir2 and cisplatin were also assessed for 24 h to compare them with the cyclometalated complex [Ir(ppy)_2_bpy]Cl^29^ (Table S2†). The ratio was defined as IC_50_ values of the complexes divided by that of cis-Pt under the same incubation conditions. The IC_50_ values and the ratio for Ir2 dramatically decreased compared to [Ir(ppy)_2_bpy]Cl (Table S2†). For example, the IC_50_ value and the ratio for complex Ir2 toward HeLa cells were 6.5 μM and 0.36, *vs.* 26.6 μM and 1.32 for [Ir(ppy)_2_bpy]Cl, respectively. Meanwhile, through the comparison of the structure and the cytotoxicity of Ir-NH_2_ and Ir2, the distinct biological behaviors of both complexes were found to have resulted from the LA ligand. The cytotoxicity of Ir2 was highly enhanced and the IC_50_ values dropped to half or one-third that of Ir-NH_2_ (Table 1). This result illuminated that the modified LA ligand enhanced the cytotoxicity of complex Ir2 and that the combination of the N^∧^N-LA ligand with the tripyridine Ir(iii) section had a positive synergistic effect. Moreover, complex Ir2 manifested a similar cytotoxicity against A549 and A549R cell lines, indicating that Ir2 could overcome the cisplatin-resistance. Reduced drug accumulation and reaction with thiol molecules are the major mechanisms in the development of resistance to cis-Pt,^30^ which has given a direction for the following mechanism studies.

### Partition coefficients (log *P*_o/w_) and cellular uptake

The different bioactivities of complexes Ir1 and Ir2 are highly related to their cellular uptake level, which is influenced by many factors, *e.g.*, molecular size, lipophilicity, water-solubility, and uptake mechanisms.^23^ The lipophilicity of complexes Ir1 and Ir2, referred to as octanol–water partition coefficients (log *P*_o/w_), was determined by using a classical shake-flask method.^31,32^ Considering the hydrolysis of Ir1, 50 mM NaCl aqueous was adopted to suppress the aquation of Ir1. The log *P*_o/w_ values for Ir1 and Ir2 were −1.06 and 1.39 (Fig. S15a†), respectively, indicating that Ir1 was hydrophilic but Ir2 was hydrophobic, which may have an effect on their cellular uptake because of the lipid bilayer of the cell membrane.

Furthermore, the cellular uptake levels of complexes Ir1 and Ir2 in A549 cells were also quantitatively determined by ICP-MS. A549 cells were pretreated with Ir1 and Ir2 for 4 h, and the iridium content in the cells was measured. The results showed 0.024 ng μg^−1^ protein for Ir2-treated A549 cells, which was about 8.5-fold that for Ir1-treated A549 cells (0.0028 ng μg^−1^ protein, Fig. S15b†). These results were consistent with the cytotoxicity evaluation, and suggested that Ir1 could be hardly taken in by A549 cells. The previously reported Ir piano stool complex [(η^5^-Cp*)Ir(N^∧^N)Cl]PF_6_ (N^∧^N = (triphenylmethyl)(pyridine-2-ylmethylene)amine) showed a similar cellular uptake in A549 cells (4.5 ng per 10^6^ cells) as complex Ir1.^27^

### Interaction with GSH and BSA

**Fig. 1:**
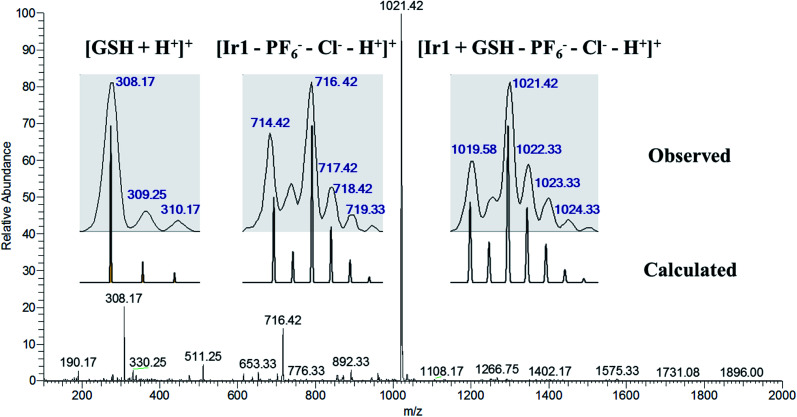
ESI-MS spectrum with the isotopic distribution of the solution sample from the reaction of Ir1 (1 mM) and GSH (10 mM) in CH_3_OH/H_2_O (v/v, 1 : 1) after incubation for 6 h at 310 K. The assignments for the peaks are listed in the inset.

Tripeptide glutathione (GSH) is an important cellular antioxidant that could bind to the metal-center of metal-arene analogues after hydrolysis and participate in the detoxification and deactivation of metal-based anticancer drugs.^30,33^ To study the interaction between the GSH and the resulting Ir1 and Ir2, titration experiments were carried out. As shown in Fig. S16a,† the absorption band at 314 nm has disappeared and the band intensity at 304 nm has gradually decreased, which suggested the structure of Ir1 could be altered and the interaction between Ir1 and GSH may happen. In addition, ESI-MS results further confirmed the existence of the Ir1 + GSH adduct (Fig. 1). The *m*/*z* peaks at 308.17 and 716.42 could be assigned to [GSH + H^+^]^+^ and [Ir1–PF_6_^−^–Cl^−^–H^+^]^+^, respectively, and the *m*/*z* peak at 1021.42 could be ascribed to the Ir1 + GSH adduct ([Ir1 + GSH–PF_6_^−^–Cl^−^–H^+^]^+^, as the major product, with the molecular GSH replacing the position of Cl^−^ and binding to the Ir(iii) center). In contrast, complex Ir2 could preserve its cyclometalated structure, where the UV-vis spectrum exhibited no obvious change (Fig. S16b†) and the ESI-MS spectrum showed only complex Ir2 species and no *m*/*z* peak for the Ir2 + GSH adduct (Fig. S17†).

Serum albumin (SA) is the main protein in blood plasma, and is important for understanding the drug pharmacokinetics and drug–protein interactions when studying the interactions between anticancer metallodrugs and human serum albumin (HSA).^25^ Bovine serum albumin (BSA) is similar to HSA and is easy to obtain; thus fluorescence quenching studies of BSA were performed to define the binding ability of the metal complexes to BSA (Fig. S18–S21†). Upon the addition of Ir1 and Ir2 into the BSA solution, the florescence intensity of BSA gradually decreased, which manifested that both Ir1 and Ir2 could interact with BSA (Fig. S18 and S20†). According to the calculations from the intercept and slope of the double-logarithm curves of the fluorescence data,^32^ the binding constants (*K*_b_) for Ir1 and Ir2 were 1.10 × 10^5^ M^−1^ and 3.69 × 10^4^ M^−1^, respectively, indicating the stronger binding ability with BSA of the metal-arene complex Ir1 than that of Ir2. Moreover, Ir2 owned more binding sites (*n*) than that of Ir1 (1.42 *vs.* 0.83) (Table S3†), suggesting Ir2 could bind to other kinds of proteins.

### Anticancer mechanism for Ir2

Complex Ir1 was almost inactive to all the tested cancer cells, which could be attributed to its low partition coefficient and cellular uptake. Thus, cyclometalated complex Ir2 was thus further chosen to study its anticancer mechanism.

**Fig. 2:**
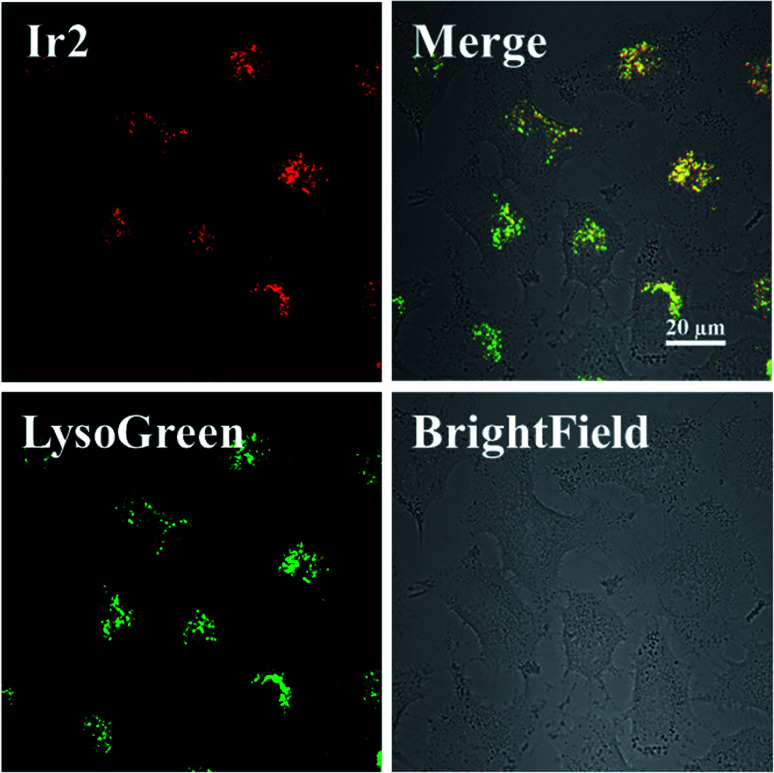
Intracellular colocalization of Ir2 with the LysoGreen probe observed by confocal microscopy. A549 cells were incubated with Ir2 (10 μM, 4 h) and then stained with LysoGreen (2 μM, 30 min). Scale bar: 20 μm.

The subcellular localization of Ir2 in A549 cells was investigated by confocal laser scanning microscopy (CLSM). With the support of the strong fluorescence of Ir2, the confocal images of Ir2-pretreated A549 cells (10 μM) over 4 h at 310 K were examined, which were then stained with commercial LysoGreen, MitoGreen, and ERGreen probes (Fig. 2 and S22†). The Pearson's correlation coefficients of the confocal images, obtained for the lysosome, mitochondria, and endoplasmic reticulum, were 0.86, 0.63, and 0.59, respectively. These results indicated that Ir2 could relatively localize and accumulate more in the lysosome of A549 cancer cells.

Apoptosis is the main cell death pathway for metal-based anticancer agents, which could inhibit the survival and division of cancer cells.^34,35^ To investigate whether Ir2 could inhibit the growth of cancer cells by apoptosis induction, A549 cells were exposed to Ir2 at different concentrations for 24 h and then measured by flow cytometry. No obvious dose-dependent apoptosis was observed toward A549 cells, even when the concentration of complex Ir2 was raised to three-fold its IC_50_ value (Fig. S23†). The proportion of necrotic cells, however, was raised from 3.35% to 11.3% for the treated A549 cells, which suggested that complex Ir2 may induce the death of some A549 cancer cells through necrosis.^36^

**Fig. 3:**
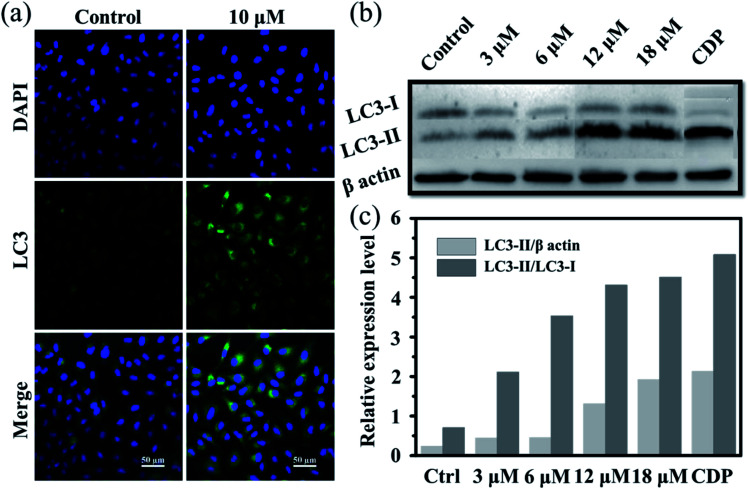
(a) Immunofluorescence imaging of LC3 in A549 cells pretreated with Ir2 (10 μM) for 24 h. Scale bar: 50 μm. (b) Protein expression of LC3 in A549 cancer cells. A549 cells were cultured with the indicated concentrations of Ir2 for 24 h and then were subjected to western blotting. (c) Relative expression level of LC3 according to the quantitative results of WB. CDP represents chloroquine diphosphate, which is a positive autophagy inducer.

Moreover, it is reported that excess autophagy can act as a pro-death mechanism, which leads to the destruction of cancer cells.^37,38^ Thus, the alternate cell death pathway, autophagic cell death, was also investigated. As is known, the expression level of LC3 protein is generally the autophagosomal marker.^39^ Immunofluorescence assessment of LC3 showed that the fluorescence intensity and dots increased in Ir2-treated A549 cells after incubation for 24 h (Fig. 3a), manifesting that vacuoles of autophagosomes emerged. Further evidence was collected from the western blotting analysis, whereby the expression of LC3-II protein increased with the increasing dose of Ir2 over 24 h in A549 cells (Fig. 3b), and LC3-I to LC3-II-conversion could be markedly seen from the histogram (Fig. 3c), which was slightly lower than that induced by chloroquine diphosphate, an autophagy inducer. These results suggested the occurrence of the autophagic cell death of A549 cells instead of apoptosis.

**Fig. 4:**
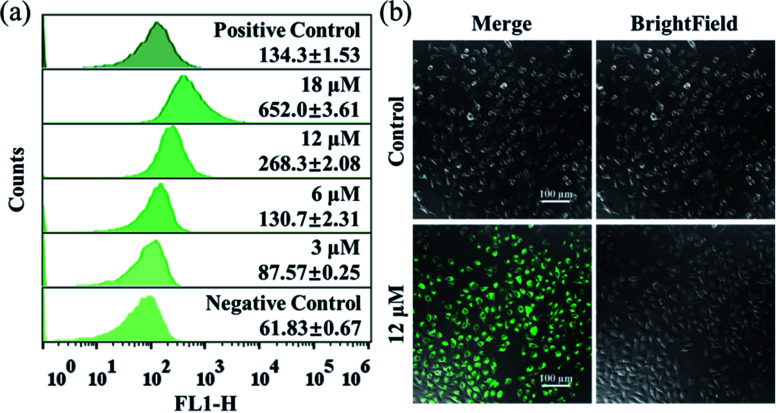
(a) Intracellular ROS analysis of A549 cancer cells exposed to Ir2 at the indicated concentrations for 24 h by using the DCFH-DA fluorescence probe (*λ*_ex_ = 488 nm; *λ*_em_ = 525 ± 20 nm) with flow cytometry. (b) Observation of ROS generated in A549 cancer cells caused by Ir2 (12 μM, 24 h) using the commercial DCFH-DA probe (10 μM, 30 min) with confocal microscopy. Scale bar: 100 μm.

The excessive generation of ROS induced by metal-based anticancer agents is usually of great importance for their anticancer MoA.^40,41^ The ROS generated in A549 cancer cells was detected by using the fluorescent probe DCFH-DA. After treatment with different concentrations of Ir2 for 24 h, the ROS level in A549 cells measured by flow cytometry was obviously elevated in a dose-dependent manner (Fig. 4a). The ROS induced by 6 μM (1× IC_50_) Ir2 was equivalent to that induced by the commercial ROS inducer. This result was further confirmed by the confocal images, whereby when A549 cells were exposed to Ir2 (12 μM) for 24 h, a significant increase in the mean fluorescence intensity (MFI) was produced compared with untreated cells (Fig. 4b). Thus, complex Ir2 was able to effectively induce the generation of intracellular ROS and subsequent cell death.

**Fig. 5:**
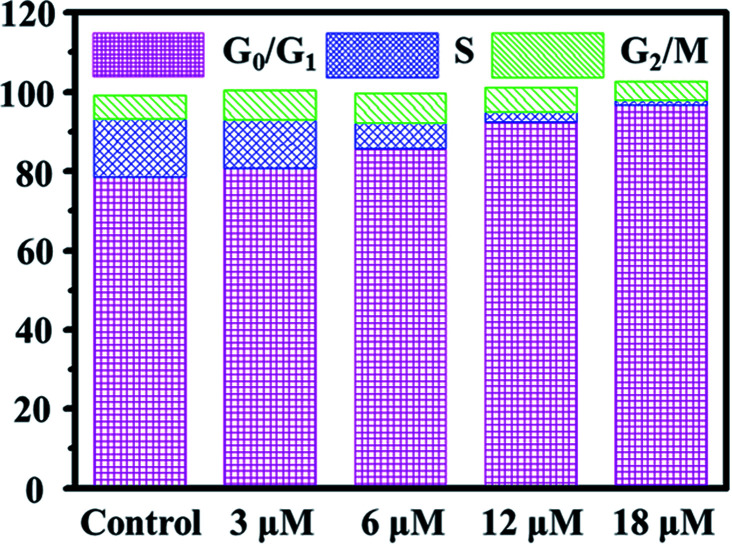
Effects of Ir2 on the A549 cell cycle distribution analyzed by flow cytometry. A549 cells were stained by PI after treatment with Ir2 at the indicated concentrations for 24 h.

Cell cycle regulation is one of the effective therapeutic methods, and anticancer agents can arrest cancer cells at a certain checkpoint due to the inhibition of cancer cell proliferation.^27,28^ Here, flow cytometric studies revealed that the mode of cell cycle arrest in A549 cells induced by Ir2 was in a concentration-dependent manner (Fig. 5 and S24†). Upon the exposure of A549 cells to complex Ir2 at 18 μM, the percentage of cells in the G_0_/G_1_ phase increased from 78.57% to 96.69%, indicating that the complex arrested the cell cycle of A549 cells at the G_0_/G_1_ phase. Hence, Ir2 could localize and accumulate in the lysosomes of A549 cells, induce ROS production, arrest the cell cycle at G_0_/G_1_, and lead to cell death by autophagy instead of apoptosis, once A549 cancer cells were exposed to complex Ir2.

## Discussion

The structures of the organometallic anticancer agents, which consist of metal centers, auxiliary ligands, and their spatial arrangements, determine their biological properties.^42^ In this work, complexes Ir1 and Ir2 with the same metal center iridium and modified N^∧^N-LA ligand exhibited apparently different bioactivities toward various cancer cell lines *in vitro*, which was ascribed to the structural diversity of complexes Ir1 and Ir2. The metal–halogen bond in the half-sandwich complex Ir1 was easy to hydrolyze,^42^ and the unstable Ir–Cl could interact with other biological molecules before reaching its target, such as GSH here. It was reported that GSH could activate osmium prodrugs with an azo group to exert their anticancer activity.^43,44^ However, after GSH attached to Ir(iii) center, Ir1 could be deactivated and the resistance could be enhanced according to the cytotoxicity. Cyclometalated Ir2 was stable in aqueous solution and underwent no reaction with GSH, which could guarantee the bioactivity of Ir2. Furthermore, the excellent photophysical property of Ir2 provided a more convenient way to study its anticancer MoA through confocal spectroscopy, such as the subcellular localization.

It is known that the cytomembrane of eukaryotic cells is a phospholipid bilayer; thus strong lipophilic molecules could enter cells freely.^45^ When the N^∧^N-LA ligand was conjugated to the dinuclear precursor [Ir(ppy)_2_Cl]_2_ forming the cyclometalated complex Ir2, the lipophilicity and cellular uptake level were greatly improved compared with that of the half-sandwich complex Ir1, which was correlated with their biological activities. The high cellular accumulation and non-reaction with GSH may be the main reasons for overcoming the cisplatin-resistance.

Compared to the traditional cisplatin reacting with DNA in the nucleus and leading to cell death through apoptosis, most Ir(iii)-based cancer agents, as previously reported, also underwent the apoptosis death path;^25^ whereas cyclometalated Ir2 could localize and accumulate in the lysosomal organelle of A549 cancer cells and induce partial necrosis and autophagic cell death, which is distinct from the apoptosis induced by LA itself. These results demonstrated that Ir2 with the functionalized N^∧^N-LA ligand could be utilized in cancer treatment due to its promising anticancer activity.

## Conclusions

In conclusion, this work utilized the emerging transition metal Ir(iii) and promising clinical drug α-lipoic acid derivative to construct two metallodrugs with half-sandwich (Ir1) and cyclometalated (Ir2) structures. Systematic studies on the biological activities of both Ir1 and Ir2 were performed. The half-sandwich complex Ir1 was deemed to be inactive, with IC_50_ values > 200 μM, which may result from its worse stability and low lipophilicity for decreased cellular uptake. Nevertheless, the cyclometalated complex Ir2 showed outstanding anti-cancer activity toward diverse cancer cell lines *in vitro* and a promising ability to overcome cisplatin-resistance in A549R cells, resulting from its better stability, lipophilicity, and cellular uptake. Ir2 demonstrated that it could localize and accumulate in subcellular lysosomes of A549, resulting in the autophagy of A549 cells instead of apoptosis. Ir2 could also induce the production of a large number of ROS species in A549 cells, and arrest the cell cycle at the G_0_/G_1_ phase to prevent the growth of A549 cells. These results illustrated that cyclometalated Ir(iii) complexes with LA and its derivatives could be potent candidates to extend the boundary of established anticancer drugs. This work has also provided insights to clarify the subtle structure–property relationship between the half-sandwich and cyclometalated complexes, and highlighted the importance of the tailored design of metallodrugs according to their future functions.

## Conflicts of interest

There are no conflicts to declare.

## Supplementary Material

RA-010-C9RA10357K-s001
