# Supplementary material for: Unveiling the anti-cancer mechanism for half-sandwich and cyclometalated Ir(iii)-based complexes with functionalized α-lipoic acid
Source: RSC Adv. 2020 Feb 3;10(9):5392–8. doi: 10.1039/c9ra10357k (PMC9049077; doi:10.1039/c9ra10357k)
Supplement: RA-010-C9RA10357K-s001 [file RA-010-C9RA10357K-s001.pdf]

Electronic Supplementary Material (ESI) for RSC Advances.
This journal is © The Royal Society of Chemistry 2020

Supplementary information

Unveiling the Anti-cancer Mechanism for Half-sandwich and Cyclometalated Ir(III)-based Complexes with Functionalized α-Lipoic Acid

Meng-Meng Wang†, Xu-Ling Xue†,Xi-Xi Sheng, Yan Su, Ya-Qiong Kong, Yong Qian, Jian-Chun Bao, Zhi Su* and Hong-Ke Liu*

Jiangsu Collaborative Innovation Center of Biomedical Functional Materials, College of Chemistry and Materials Science, Nanjing Normal University, Nanjing, 210023, China.

*Email: zhisu@njnu.edu.cn; liuhongke@njnu.edu.cn

† The authors contributed equally.

Experimental section

Materials and measurements

α-lipoic acid (HEOWNS), EDCI (N-(3-Dimethylaminopropyl)-3-ethylcarbodiimide hydrochloride, HEOWNS), HOBt (1-Hydroxybenzotriazole, HEOWNS), Iridium(III) chloride hydrate (J&K Scientific), ppy (2-Phenylpyridine, HEOWNS), NH_4PF_6 (HEOWNS), cisplatin (Energy Chemical), GSH (Glutathione reduced, Energy Chemical), BSA (Bovine Serum Albumin, Biosharp), DMSO (dimethyl sulfoxide, Sigma Aldrich), MTT (3-(4,5-dimethylthiazol-2-yl)-2,5-diphenyltetrazolium bromide, HEOWNS), DMEM (Dulbecco's modified Eagle's medium, Gibco BRL) or RPMI 1640 (Roswell Park Memorial Institute medium 1640, Gibco BRL) medium containing 10% FBS (fetal bovine serum, Gibco BRL), 100 μg/mL streptomycin, and 100 U/mL penicillin (Gibco BRL), MitoGreen (Mito Tracker Green, KeyGEN BioTECH, China), LysoGreen (KeyGEN BioTECH, China), ER Green (KeyGEN BioTECH, China), DAPI (KeyGEN BioTECH, China), Annexin V-FITC apoptosis detection kit (KeyGEN BioTECH, China), Autophagy detection kit (KeyGEN BioTECH, China), LC3 Polyclonal ANTIBODY (Proteintech, USA), Fluorescein (FITC)-conjugated Affinipure Goat Anti-Rabbit lgG(H+L) (Proteintech, USA), RIPA lysis buffer (Beyotime Biotechnology, China), BCA Protein Quantitation Assay (KeyGEN BioTECH, China), DCFH-DA (2',7'-dichlorodihydrofluorescein diacetate, KeyGEN BioTECH, China), Cell Cycle Detection Kit (KeyGEN BioTECH, China). Deuterated solvents for NMR purposes were obtained from Merck and Cambridge Isotopes. Other organic reagents, which were of analytical grade, were obtained from domestic chemical corporations and used as received without any further purification.

^1H NMR spectra were recorded on a Bruker AVANCE 400 spectrometer at ambient temperature. Electrospray ionization mass spectra (ESI-MS) were obtained using an LCQ spectrometer (Thermo Scientific). UV/Vis spectra were recorded on a LAMBDA 365 UV-vis spectrophotometer. Emission spectra were measured using a FS5 Spectrofluorometer. Cells used in this work were incubated in a humidified incubator (BB 150 CO_2 incubator, Thermo Fisher Scientific, USA). MTT data were recorded on a microplate reader (LabServ K3, Part of Thermo Fisher Scientific, USA). Cell imaging experiments were carried out on a confocal microscope (A1, Nikon, Japan). Flow cytometric analysis was done using a flow cytometer (BD FACSverse, USA). Western blotting experiments were conducted on Mini-Protean Tetra System (BIO RAD, Power Pac^{TM} HC, Sinapore). The western

blotting signal was enhanced by Tanon High-sig ECL Western Blotting Substrate and visualized by Tanon 5200 Multi.

Synthesis and characterization

Synthesis of the ligand (N^N-LA): The modified α-lipoic acid ligand (N^N-LA) was synthesized from 4-Aminomethyl-4-methyl-2,2-bipyridyl, which was prepared in a slightly modified way according to literature procedures.[1, 2] To a suspension of α-lipoic acid (0.516 g, 2.5 mmol), EDCI (0.479 g, 2.5 mmol), HOBt (0.270 g, 2 mmol) in DMF, 4-Aminomethyl-4-methyl-2,2-bipyridyl (0.498 g, 2.5 mmol) and 2 mL trimethylamine were added in a 50 mL Schlenk tube. The reaction mixture was stirred under argon atmosphere at ambient temperature for 24 h, then treated with ice-cold water and a white precipitate was filtered. The solid obtained was dissolved in CH_2Cl_2 and purified by column chromatography on silica gel eluted with CH_2Cl_2/CH_3OH (100:1, v/v). Yield: 0.179 g (18%). ^{1}H NMR (400 MHz, $CDCl_3$) δ (ppm): 8.53 (dd, J = 32.9, 4.9 Hz, 2H), 8.20 (d, J = 20.6 Hz, 2H), 7.15 (dd, J = 22.9, 4.3 Hz, 2H), 6.35 (s, 1H), 4.48 (d, J = 5.9 Hz, 2H), 3.56-3.45 (m, 1H), 3.09 (ddt, J = 24.8, 11.0, 6.8 Hz, 2H), 2.41 (s, 3H), 2.24 (t, J = 7.3 Hz, 2H), 1.85 (td, J = 13.6, 6.9 Hz, 1H), 1.65 (dt, J = 13.7, 7.9 Hz, 4H), 1.49-1.39 (m, 2H), 1.23 (s, 1H). ^{13}C NMR (101 MHz, $CDCl_3$) δ (ppm): 172.94, 156.61, 155.59, 149.46, 148.93, 148.45, 148.30, 124.91, 122.41, 122.12, 119.60, 56.36, 42.57, 40.21, 38.46, 36.30, 34.60, 28.87, 25.34, 21.21.

Syntheses of the complexes (**Ir1**, **Ir-NH$_2$** and **Ir2**): The two dichloro-bridged iridium dinuclear precursors $[(\eta^5\text{-Cp*})IrCl_2]_2$ and $[Ir(ppy)_2Cl]_2$ were prepared according to literature procedures.[3, 4] Complexes **Ir1** and **Ir2** were synthesized by reacting two equivalents of the modified α-lipoic acid ligand (N^N-LA) (0.1 mmol, 2 equiv.) with the corresponding dinuclear precursors (0.05 mmol, 1 equiv.) in a mixture solvent of CH_2Cl_2/CH_3OH (2:1, v/v) under the argon atmosphere respectively, which were then isolated by anion exchange with NH_4PF_6. Complex **Ir-NH$_2$** was obtained by replacing the N^N-LA ligand with 4-Aminomethyl-4-methyl-2,2-bipyridyl compound under the same reaction condition of **Ir2**. Pure products of these three complexes were obtained by recrystallizing from CH_2Cl_2/CH_3OCH_3.

[Ir(Cp*)(N^N-LA)Cl]PF_6 (**Ir1**): Complex **Ir1** was obtained as a yellow powder. Yield: 0.048 g (54%).

^1H NMR (400 MHz, d_6-DMSO) δ (ppm): 8.88 (d, J = 5.9 Hz, 1H), 8.81 (d, J = 5.8 Hz, 1H), 8.64-8.57 (m, 3H), 7.69 (d, J = 4.9 Hz, 1H), 7.64 (d, J = 5.8 Hz, 1H), 4.54 (dd, J = 15.2, 8.7 Hz, 2H), 3.61 (dt, J = 15.2, 5.9 Hz, 1H), 3.22-3.07 (m, 2H), 2.64 (s, 3H), 2.41 (dtd, J = 14.6, 6.4, 2.3 Hz, 1H), 2.26 (t, J = 7.3 Hz, 2H), 1.90-1.80 (m, 1H), 1.69-1.55 (m, 19H), 1.40 (dd, J = 15.4, 7.7 Hz, 2H). ^{13}C NMR (101 MHz, d_6-DMSO) δ (ppm): 173.20, 155.12, 154.80, 154.44, 152.90, 152.15, 151.67, 130.00, 126.97, 125.04, 122.59, 99.99, 89.28, 56.63, 41.69, 38.59, 35.52, 34.59, 28.85, 25.41, 21.26, 8.61. ESI-MS (CH_3OH): calcd for [**Ir1**-PF_6^-]$^+$ m/z = 750.47, found m/z = 750.50; calcd for [**Ir1**-PF_6^--Cl^-]$^{2+}$ m/z = 357.51, found m/z = 357.75.

[Ir(ppy)$_2$(N^N-NH_2)](PF_6) (**Ir-NH$_2$**): Complex **Ir-NH$_2$** was obtained as a yellow powder. Yield: 0.049 g (58%). ^1H NMR (400 MHz, d_6-DMSO) δ (ppm): 8.81 (s, 1H), 8.75 (s, 1H), 8.27 (d, J = 7.8 Hz, 2H), 7.93 (t, J = 8.0 Hz, 4H), 7.74 (d, J = 5.4 Hz, 1H), 7.69 (d, J = 5.5 Hz, 1H), 7.65 – 7.59 (m, 3H), 7.53 (d, J = 5.3 Hz, 1H), 7.17 (t, J = 6.5 Hz, 2H), 7.02 (t, J = 7.4 Hz, 2H), 6.90 (t, J = 7.4 Hz, 2H), 6.20 (d, J = 7.5 Hz, 2H), 3.94 (s, 2H), 2.54 (s, 3H). ^{13}C NMR (101 MHz, d_6-DMSO) δ (ppm): 167.35, 167.33, 157.56, 155.54, 155.48, 151.99, 151.27, 151.25, 149.57, 149.52, 149.20, 149.11, 144.27, 139.16, 131.56, 130.68, 129.69, 127.21, 125.90, 125.53, 124.37, 124.33, 123.45, 122.63, 120.50, 120.47, 44.90, 21.42. ESI-MS (CH_3OH): calcd for [**Ir-NH$_2$**-PF_6^-]$^+$ m/z = 699.86, found m/z = 700.50

[Ir(ppy)$_2$(N^N-LA)](PF_6) (**Ir2**): Complex **Ir2** was obtained as a yellow powder. Yield: 0.062 g (60%). ^1H NMR (400 MHz, d_6-DMSO) δ (ppm): 8.71 (d, J = 9.9 Hz, 2H), 8.48 (t, J = 5.8 Hz, 1H), 8.26 (dd, J = 8.0, 3.3 Hz, 2H), 7.93 (ddd, J = 11.2, 6.5, 2.2 Hz, 4H), 7.78 (d, J = 5.7 Hz, 1H), 7.70 (d, J = 5.6 Hz, 1H), 7.61 (t, J = 6.1 Hz, 2H), 7.54 (d, J = 5.1 Hz, 1H), 7.48 (d, J = 5.5 Hz, 1H), 7.20-7.13 (m, 2H), 7.02 (t, J = 7.5 Hz, 2H), 6.90 (t, J = 7.4 Hz, 2H), 6.19 (dd, J = 7.1, 2.6 Hz, 2H), 4.47 (dd, J = 15.0, 8.8 Hz, 2H), 3.58 (dd, J = 8.7, 5.7 Hz, 1H), 3.21-3.03 (m, 2H), 2.53 (d, J = 6.5 Hz, 3H), 2.37 (td, J = 12.3, 6.0 Hz, 1H), 2.21 (t, J = 7.3 Hz, 2H), 1.84 (td, J = 13.4, 6.8 Hz, 1H), 1.58 (tdd, J = 20.5, 13.8, 7.3 Hz, 4H), 1.34 (dt, J = 14.1, 7.0 Hz, 2H). ^{13}C NMR (101 MHz, d_6-DMSO) δ (ppm): 173.13, 167.33, 155.59, 155.25, 153.51, 151.97, 151.06, 150.09, 149.56, 149.11, 144.25, 139.19, 131.54, 130.69, 129.79, 126.72, 126.01, 125.53, 124.34, 123.53, 122.66, 120.48, 56.59, 41.80, 40.02, 38.54, 35.44, 34.55, 28.76, 25.28, 21.44. ESI-MS (CH_3OH): calcd for [**Ir2**-PF_6^-]$^+$ m/z = 888.17, found m/z = 888.50.

Stability and Hydrolysis

The abilities of hydrolysis of complexes **Ir1** and **Ir2** were monitored by UV-vis spectroscopy. A solution of complexes **Ir1** and **Ir2** (50 μM) were prepared in 5% DMSO/95% H_2O (v/v) respectively. The absorbance was recorded at different time intervals in the range from 200 nm to 800 nm over 24 h at 298 K. Plot of the change in absorbance with time was fitted to the appropriate eq. ($A = C_0 + C_1 * e^{-kt}$, where C_0 and C_1 are computer-fitted constants and A is the absorbance corresponding to t) for pseudo first order kinetics to give the rate constant and half-life of hydrolysis. The stability of complexes **Ir1** and **Ir2** were also studied using ^1H NMR at different time intervals.

UV-vis Spectroscopy

A LAMBDA 365 UV-vis spectrophotometer was used with 1 cm path-length quartz cuvettes (3 mL). Stock solutions of **Ir1** and **Ir2** in DMSO were prepared before measurements, and then diluted suitably with distilled water to the required concentration. The absorption spectra were recorded at 298 K unless otherwise stated.

Fluorescence measurements

A FS5 Spectrofluorometer was used with 1 cm path-length quartz cuvettes (3 mL). Stock solutions of **Ir1** and **Ir2** in DMSO were prepared before measurements, and then diluted suitably with distilled water to the required concentration. Fluorescence spectra were obtained by recording the emission spectra (from 425 to 800 nm) at the excited wavelength of 405 nm. The fluorescence spectra were recorded at 298 K unless otherwise stated.

Cell lines and culture conditions

A549, A549R, MCF-7, A2780, HeLa and LO2 cells were obtained from Experimental Animal Center of Sun Yat-Sen University (Guangzhou, China). Cells were routinely cultured in DMEM (Dulbecco's modified Eagle's medium, Gibco BRL) or RPMI 1640 (Roswell Park Memorial Institute medium 1640, Gibco BRL) medium containing 10% FBS (fetal bovine serum, Gibco BRL), 100 μg/mL streptomycin, and 100 U/mL penicillin (Gibco BRL). The cells were cultured in tissue culture flasks in a humidified incubator (BB 150 CO_2 incubator, Thermo Fisher Scientific, USA), which provided an atmosphere of 5% CO_2 and 95% air at a constant temperature of 37 °C. Cisplatin-resistant A549R

cells were cultured in RPMI 1640 with additional cisplatin to maintain the drug resistance.

Cytotoxicity assay (MTT assay)

The cytotoxicity of LA, N^N-LA and complexes synthesized towards various cancer cell lines was determined by MTT assay. After plating 5000 cells per well in 96-well plates, the cells were pre-incubated in drug-free media for 24 h at 37 °C to grow to confluence. The complexes were dissolved in DMSO (1%, v/v) just before the experiments, and diluted with fresh media immediately. Then, cells were incubated with a series of different concentrations of the tested complexes for 44 h at 37 °C. After that, 20 μL of MTT solution (5 mg/mL) was added to each well, and the plates were incubated for an additional 4 h. The medium was carefully removed and 150 μL DMSO was added per well. The plates were incubated for 10 min with shaking. The absorbance at 595 nm was measured using a microplate reader (LabServ K3, Part of Thermo Fisher Scientific, USA). Each well was triplicated to gain the mean values. IC_{50} values quoted are mean ± SEM.

Lipophilicity

The lipophilicity of complexes **Ir1** and **Ir2**, which was presented as octanol-water partition coefficient (Log $P_{o/w}$), was determined using a classical shake-flask method.[5] Octanol-saturated water (OSW) and water-saturated octanol (WSO) were prepared by shaking analytical grade octanol and 50 mM NaCl aqueous solution (to suppress hydrolysis of the chloride complex) together using a laboratory shaker for 24 h to allow saturation of both phases. Stock solutions of complexes were prepared in the aqueous phase at a concentration of 100 μM, and then were added to an equal volume of saturated 1-octanol phase. The resultant biphasic solutions were mixed for 4 h at 500 rpm at 310 K, and then centrifuged (3000 rpm, 5 min) to separate the phases. The concentrations of the complexes in the organic and aqueous phases were determined by UV-vis absorbance at 260 nm. Log $P_{o/w}$ was defined as the logarithm of the ratio of the concentrations of the complex in the organic and aqueous phases (Log $P_{o/w}$ = Log {[Au(org)]/[Au(aq)]}; values reported are the means of three separate determinations).

ICP-MS measurement

The cellular uptake capacity of the complexes was measured by determination of intracellular iridium contents. Briefly, A549 cells were seeded in a 6-well plate at a density of 2 × 10^5 cells/well and

incubated overnight under standard growth condition. The culture medium was removed and replaced with fresh medium/DMSO (v/v, 99:1) containing complex **Ir1** and **Ir2** (10 μM). After incubation for 4 h, the cells were harvested with trypsin and washed with PBS buffer (1X) for three times. Cell pellets were collected by centrifugation and then digested with concentrated nitric acid (100 μL) at 95 °C for 2 h, hydrogen peroxide (30%, 50 μL) at 95 °C for 1.5 h, and concentrated hydrochloric acid (50 μL) at 95 °C for 1.5 h to give fully homogenized solutions. Finally, the solutions were diluted with MiliQ water to a final volume of 2 mL, and Ir contents in the samples were determined by ICP-MS (X Series 2, Thermo Fisher, USA). The average of three parallel experimental data was reported as the final result.

Interaction with GSH

The reaction of complexes **Ir1** and **Ir2** (30 μM) with different ratio of GSH (r = 0.5, 1, 1.5, 2) in 5% CH_3OH/95% H_2O (v/v) was monitored by UV-vis at 298 K. Before mixture, a series of stock solutions of **Ir1** and **Ir2** (2 mM) in CH_3OH and GSH (10 mol equiv) in H_2O were prepared respectively. An equal volume of CH_3OH containing corresponding complex and H_2O containing GSH were mixed, and then were incubated for 6 h at 310 K for ESI-MS measurements.

Study of BSA Interactions

The fluorescence emission spectrum of BSA in the absence and presence of complexes was analyzed to investigate the interaction of BSA with the complexes. The fluorescence measured in this work was calibrated to correct the "inner filter" effects (IFEs).[6] Comparing the absorption and fluorescence emission spectra of metallodrugs and BSA, it is not difficult to find that the absorption of metallodrugs show partial overlap with the absorption and fluorescence spectra of BSA. Thus both excitation light and the excitation induced emission light from BSA can be absorbed by metallodrugs, and it is not scientific to ignore the IFEs brought by metallodrugs. Relative importance of each process depends upon the absorbance of the sample at the excitation and emission wavelengths. When the absorbance of the solution is lower than 0.3, the following equation can be applied to correct the inner filter effects:

$$F_{corr} = F_{obs} \text{ antilog } [(A_{ex} + A_{em})/2]$$

where F_{obs} and F_{corr} are the measured and corrected fluorescence intensities, respectively. A_{ex} and A_{em} are the absorbances at excitation and emission wavelengths, respectively.

The titration experiments of fluorescence quenching were performed at a constant concentration of BSA. A stock solution of BSA (5 μM) was prepared in PBS buffer (5 mM) and stored at 4 °C. All spectra were recorded with excitation at 285 nm after each successive addition of the complexes and incubation at 37 °C for 5 min to complete the interaction. The Stern-Volmer quenching constant (K_{sv}) was obtained as the slope of F_0/F versus [Q] using the classical Stern-Volmer equation.[7] The binding constant K_b and number of complexes bound to BSA (n) were calculated from the intercept and slope of the double-logarithm curves of the fluorescence data.[8] Synchronous fluorescence spectra of BSA with various concentrations of complexes (0-45 μM) were obtained from 240 to 350 nm when Δλ = 15 nm and Δλ = 60 nm were set for the experiments, respectively. The spectrum with Δλ = 15 nm setting could offer the characteristic information of tyrosine, while the use of Δλ = 60 nm could highlight tryptophan residues. With the concentrations of **Ir1** and **Ir2** increasing, both spectra (Δλ = 15 nm and 60 nm) for BSA displayed a significant decrease in fluorescence intensity. However, only the red-shifting of the maximum emission for complex **Ir2** was observed (Fig. S21), while **Ir1** maintained no change (Fig. S19), indicating that the microenvironment of the two aromatic acid residues in BSA was altered upon addition of **Ir2**.

Cellular localization assay

A549 cells were seeded into 35 mm confocal dishes (JET BIOFIL, Canada) for confocal microscopy. After cultured overnight, the cells were preincubated with complex **Ir2** (10 μM) for 4 h at 37 °C. Subsequently, the medium was replaced with staining medium containing MitoGreen (200 nM), LysoGreen (2 μM), or ERGreen (2 μM) and stained for 30 min at 37 °C. The staining medium was removed and the cells were washed with PBS for three times, and then observed immediately under a confocal microscope (A1, Nikon, Japan). Complex **Ir2** was excited at 405 nm and emission was collected at 605 ± 20 nm. The excitation wavelength of MitoGreen, LysoGreen and ERGreen dyes was 488 nm and emission was collected at 516 ± 20 nm, 505 ± 20 nm and 520 ± 20 nm respectively.

Annexin V/PI double staining assay

Translocation of phosphatidylserine (PS) was measured by flow cytometry using the Annexin V-FITC apoptosis detection kit (KeyGEN BioTECH, China) according to the manufacturer's instructions. A549 cells (1.5×10^6/2 mL per well) were seeded in a 6-well plate. Cells were preincubated in drug-

free media for 24 h at 37 °C, after which complex **Ir2** was added at the indicated concentrations. After exposing to the complex for 24 h, cells were collected, washed twice with PBS buffer, and then resuspended in 500 μL binding buffer. The cell suspension in each tube was stained with 5 μL of Annexin V-FITC and 5 μL of Propidium Iodide at room temperature for 15 min in the dark. Subsequently, the tubes were placed in an ice bath in the dark. The samples were analyzed by a flow cytometer (BD FACSverse, USA). Data were analyzed using FlowJo 7.6.1 software. 10, 000 cells were acquired for each sample.

Autophagic Cell Death

Western blotting. A549 cells were treated with different concentrations of **Ir2** for 24 h at 37 °C. Subsequently, the cells were collected and treated with RIPA lysis buffer (Beyotime Biotechnology, China) for 30 min on ice. The lysate was centrifuged (13400 rmp, 4 °C) for 20 min and the precipitate was disposed. The expression level of proteins was determined according to BCA Protein Quantitation Assay (KeyGEN BioTECH, China). Then, SDS-PAGE Sample Loading Buffer (6×) was added and the samples were heated at 95 °C for 6 min, then stored at -20 °C. 20 μg of proteins were analyzed by Western blotting (12% acrylamide gel for LC3). The PVDF membranes (Darmstadt, Germany) were used to transfer proteins (200 mA, 1 h). After transmembrane, the PVDF membranes were blocked with skim milk (5% in PBST) and then incubated with primary LC3 antibody (Proteintech, USA) at 4 °C overnight and HRP-conjugated secondary antibody (Proteintech, USA) at room temperature for 1 h. The signal was enhanced by Tanon High-sig ECL Western Blotting Substrate and visualized by Tanon 5200 Multi.

Immunofluorescence assay. For the LC3 assay, A549 cells were seeded into 35 mm confocal dishes (JET BIOFIL, Canada) for confocal microscopy. After cultured overnight, the cells were treated with complex **Ir2** (10 μM) for 24 h at 37 °C. Then A549 cells were fixed with 3.7% paraformaldehyde/PBS for 15 min at the room temperature. After washed with PBS for three times, cells were then stained with anti-LC3 antibody (1: 100) for 1 h at the room temperature, and detected with a secondary antibody (Fluorescein (FITC)-conjugated affinipure goat anti-rabbit lgG(H+L), 1: 50). The cells were counterstained with 300 nM DAPI for 5 min and immediately imaged by confocal microscopy (A1, Nikon, Japan).

ROS generation assay

Flow cytometry. Flow cytometry analysis of ROS generation in A549 cells caused by exposure to complex **Ir2** was carried out using the ROS Detection Kit (KeyGEN BioTECH, China) according to the supplier's instructions. A549 cells (1.5 × 10^6/2 mL per well) were seeded in a 6-well plate. Cells were preincubated in drug-free media for 24 h at 37 °C, and then complex **Ir2** was added at the indicated concentrations. After 24 h of drug exposure, cells were incubated with the DCFH-DA probe (10 μM) for 30 min at 37 °C in the dark. Then, cells were collected by centrifugation and washed twice with serum-free medium to remove the excess staining dye. The ROS analysis was immediately performed by flow cytometry (BD FACSverse, USA) with excitation at 488 nm and emission at 530 ± 30 nm. Data were analyzed using FlowJo 7.6.1 software. 10, 000 cells were acquired for each sample.

Confocal microscopy. A549 cells were seeded into 35 mm confocal dishes (JET BIOFIL, Canada) for confocal microscopy. After cultured overnight, the cells were treated with complex **Ir2** (12 μM) for 24 h at 37 °C. After stained with 10 μM DCFH-DA probe and washed twice with serum-free medium, the cells were immediately observed by confocal microscopy (A1, Nikon, Japan) with excitation at 488 nm and emission at 530 ± 20 nm.

Cell cycle analysis

Cell cycle distribution of A549 cells induced by complex **Ir2** was carried out using the Cell Cycle Detection Kit (KeyGEN BioTECH, China) according to the supplier's instructions. A549 cells (1.5 × 10^6/2 mL per well) were seeded in a 6-well plate. Cells were preincubated in drug-free media for 24 h at 37 °C, and then complex **Ir2** was added at the indicated concentrations. After 24 h of drug exposure, the cells were collected by centrifugation and washed twice with PBS. Then, cells were fixed in 70% cold ethanol (1 mL). After storage at -20 °C overnight, cells were centrifuged and washed twice with cold PBS, and then stained with 500 μL PI/RNase A(9: 1, v/v) in the dark for 30 min at the room temperature. The cells were washed and resuspended in PBS before being analyzed by a flow cytometry (BD FACSverse, USA) with excitation at 488 nm and emission at 585 ± 30 nm. Data were processed using FlowJo 7.6.1 software. 10, 000 cells were acquired for each sample. The cell cycle distribution is shown as the percentage of cells containing G_0/G_1, S and G_2/M DNA as identified by propidium iodide staining.

Table S1. Hydrolysis data for complex **Ir1**.

Complex	Extent (%)*	k (h^{-1})	$t_{1/2}$ (h)
**Ir1**	16	0.093	7.5

*extent represents the ratio of the hydrolyzed complex.

Table S2. IC_{50} values of complexes **Ir2** and **[Ir(ppy)$_2$bpy]Cl** towards several cancer cell lines.[a]

Complex	IC_{50} (μM)					
	A549	*Ratio*[c]	MCF-7	*Ratio*	HeLa	*Ratio*
**Ir2**	8.47±0.44	0.38	6.20±0.30	0.26	6.47±0.37	0.36
***cis*-Pt**	22.39±1.50		23.51±0.82		17.94±0.47	
**[Ir(ppy)$_2$bpy]Cl**[b]	22.2±0.8	0.58	22.7±3.2	0.72	26.6±1.4	1.32
***cis*-Pt**[b]	38.6±0.9		31.4±3.0		20.2±1.8	

[a]IC_{50} values were given in μM, and cisplatin (*cis*-Pt) was included for comparison. Data are presented as mean values ± standard deviations. Cell viability was assessed after 24 h incubation. [b]IC_{50} values of **[Ir(ppy)$_2$bpy]Cl** and *cis*-Pt were cited from the reference 9, which was incubated for 24 h. [c]*Ratio* was referred as IC_{50} values of complexes **Ir2** or **[Ir(ppy)$_2$bpy]Cl** divided by that of *cis*-Pt, respectively.

Table S3. Values of K_{sv}, K_q, K_b, and n for complexes **Ir1** and **Ir2** at 310 K.

System	K_{sv}/(M^{-1})	K_q/($M^{-1}s^{-1}$)	K_b/(M^{-1})	n
**Ir1-BSA**	5.95×10^{4}	5.95×10^{12}	1.10×10^{5}	0.83
**Ir2-BSA**	2.12×10^{5}	2.12×10^{13}	3.69×10^{4}	1.42

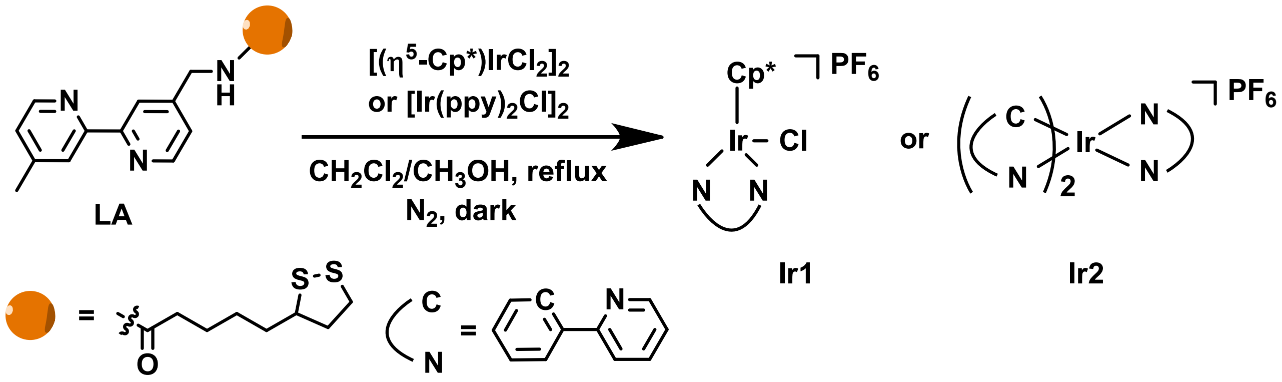

Scheme S1. Synthesis of complexes **Ir1** and **Ir2**.

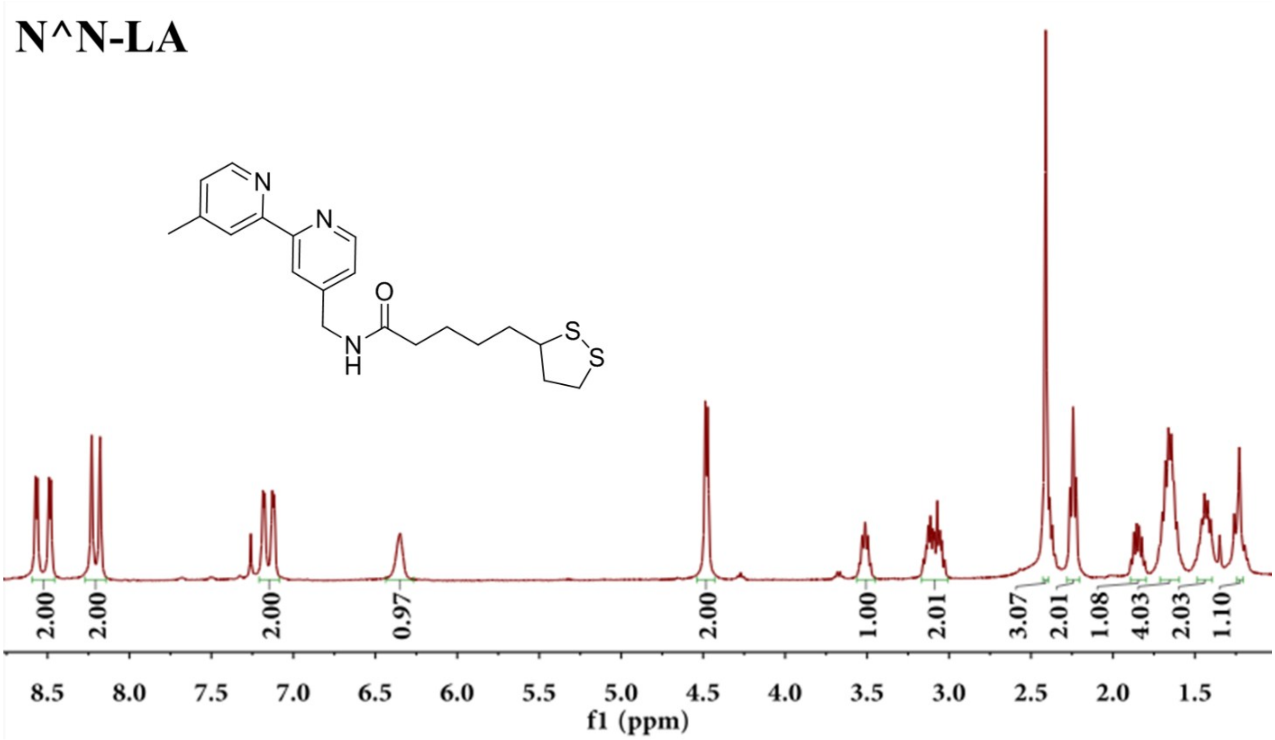

Fig. S1 ^{1}H NMR spectrum (400 MHz, $CDCl_3$) of the modified ligand N^N-LA.

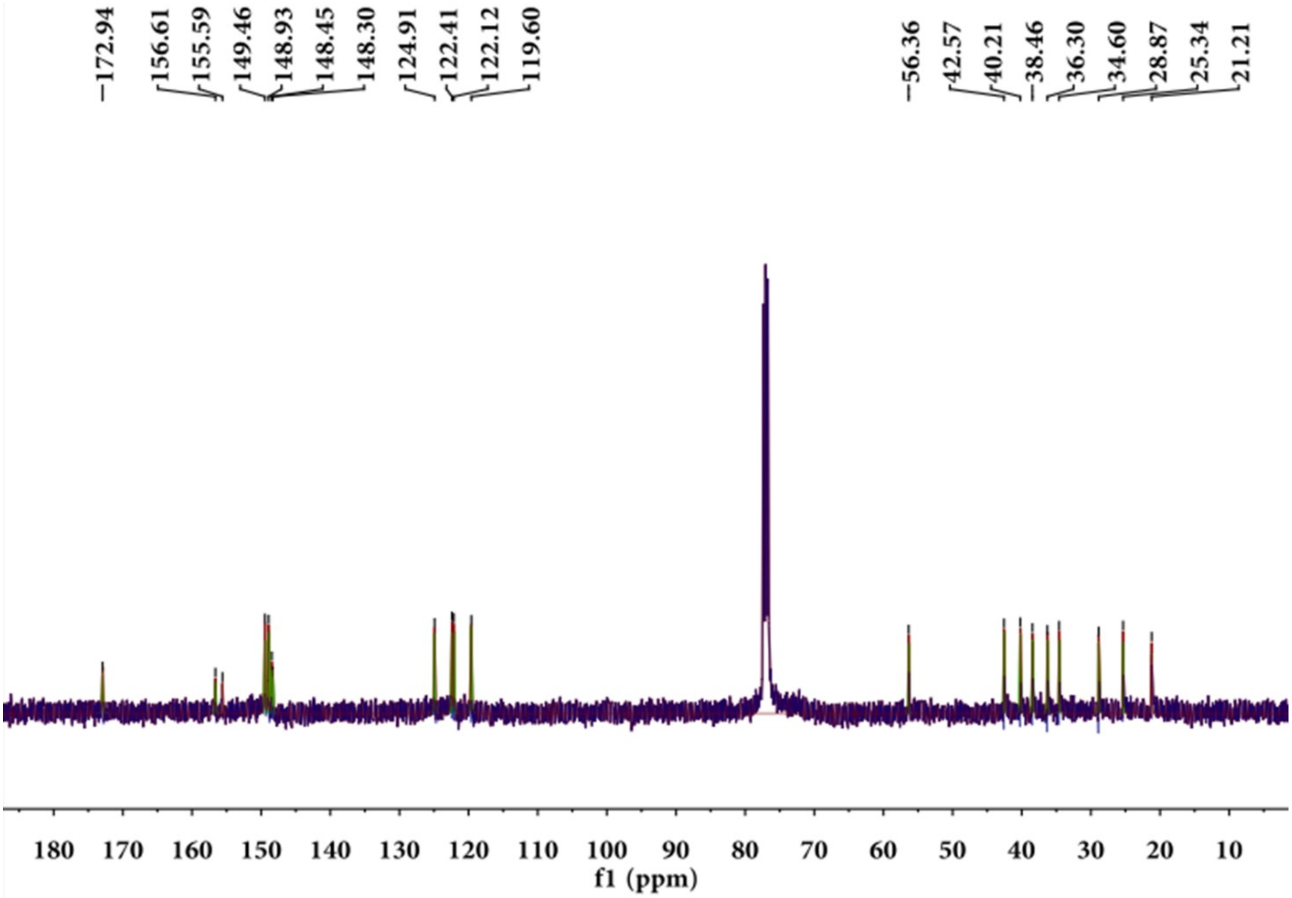

Fig. S2 ^{1}C NMR spectrum (101 MHz, $CDCl_3$) of the modified ligand N^N-LA.

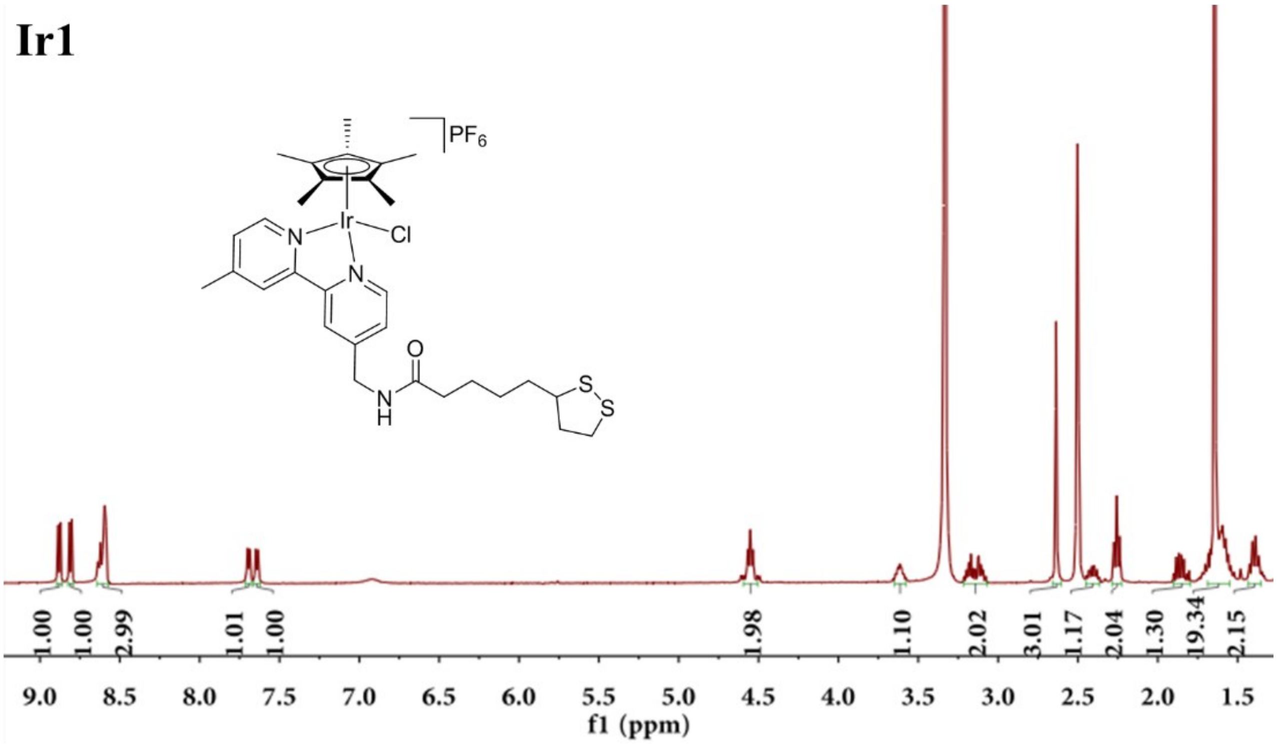

Fig. S3 ^{1}H NMR spectrum (400 MHz, d_6-DMSO) of complex **Ir1**.

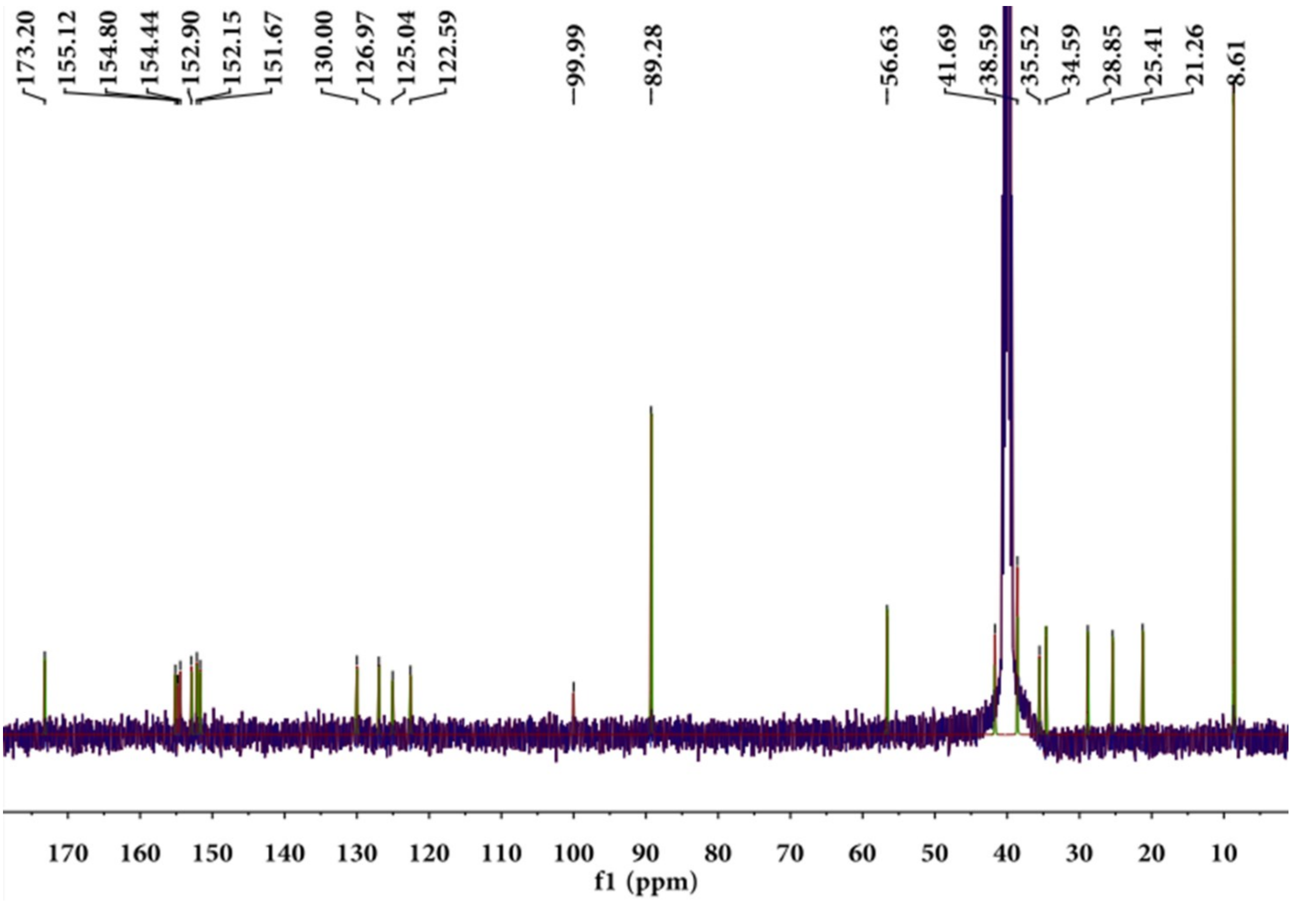

Fig. S4 [1]C NMR spectrum (101 MHz, d_6-DMSO) of complex **Ir1**.

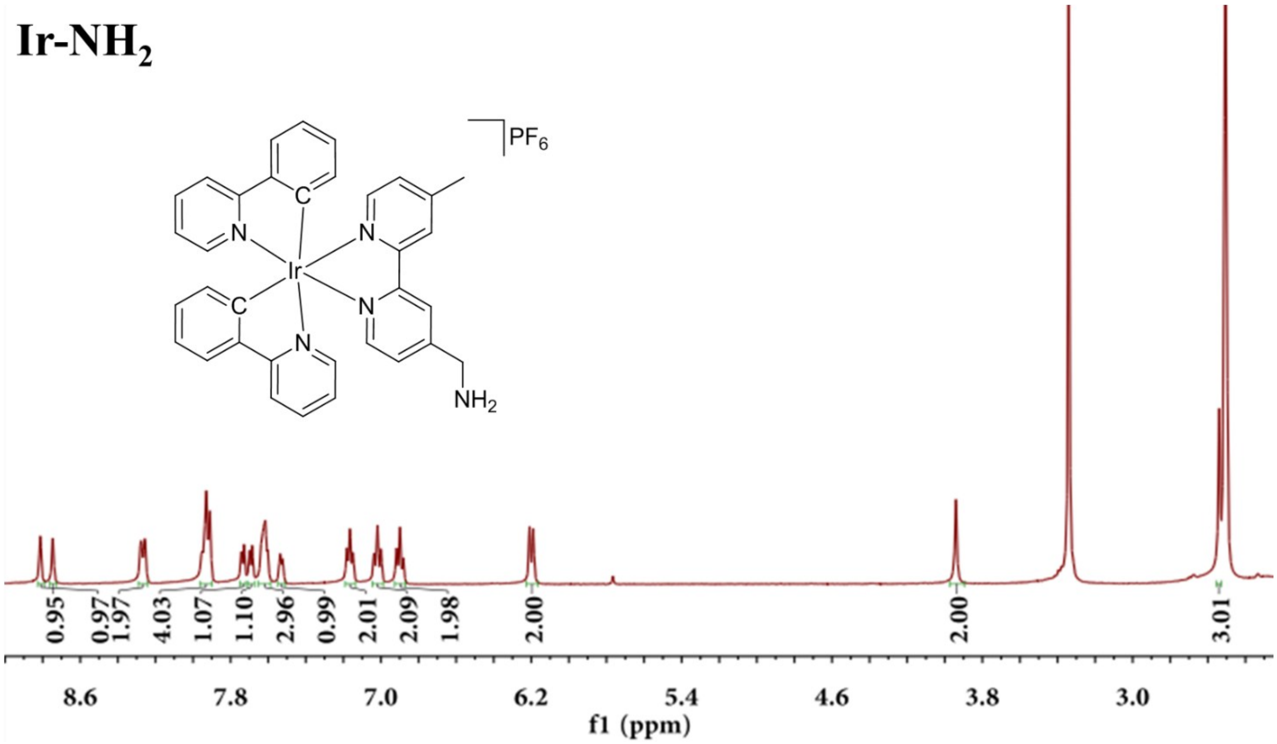

Fig. S5 ^{1}H NMR spectrum (400 MHz, d_6-DMSO) of complex **Ir**-**NH$_2$**.

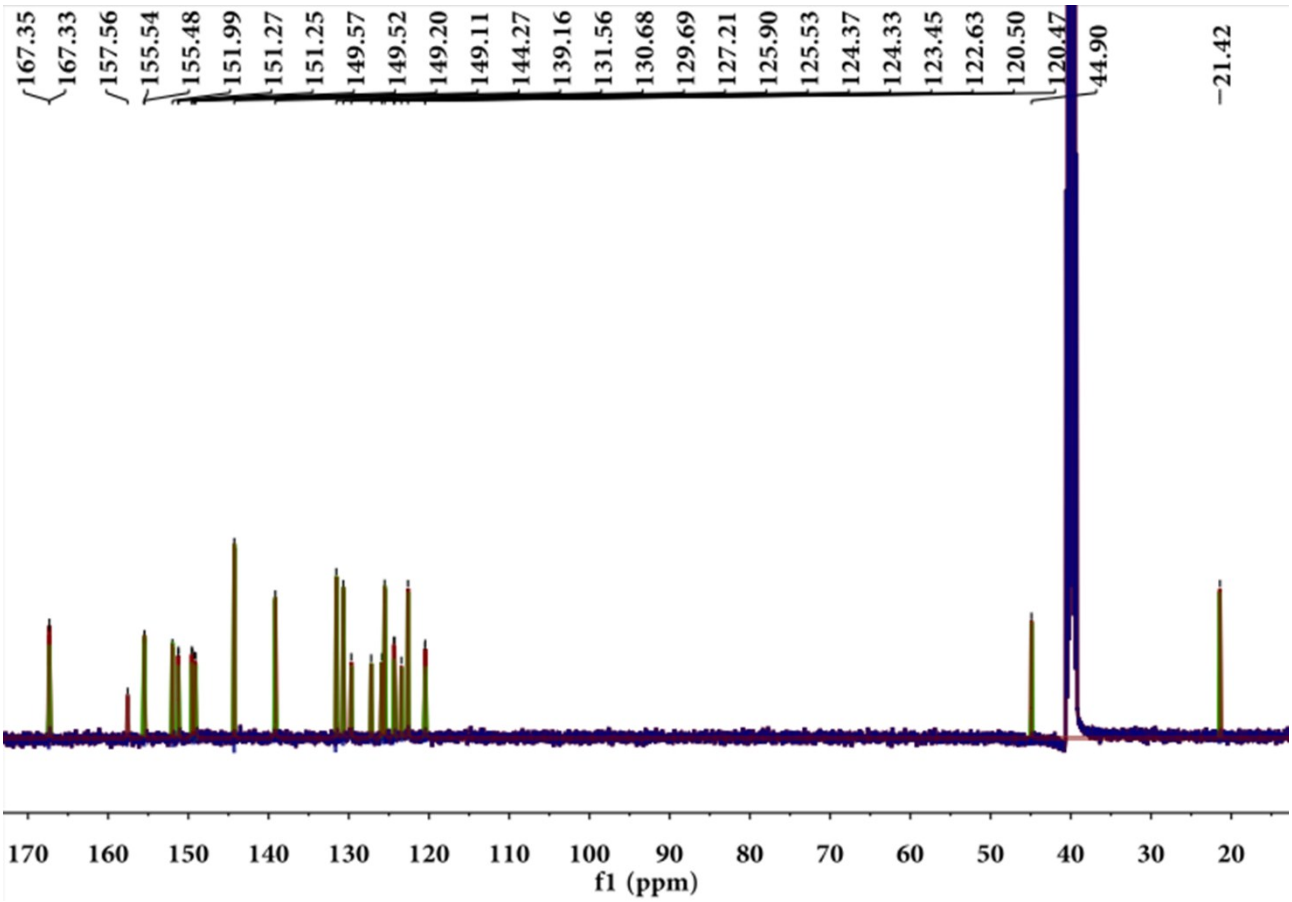

Fig. S6 [1]C NMR spectrum (101 MHz, d_6-DMSO) of complex **Ir**-**NH$_2$**.

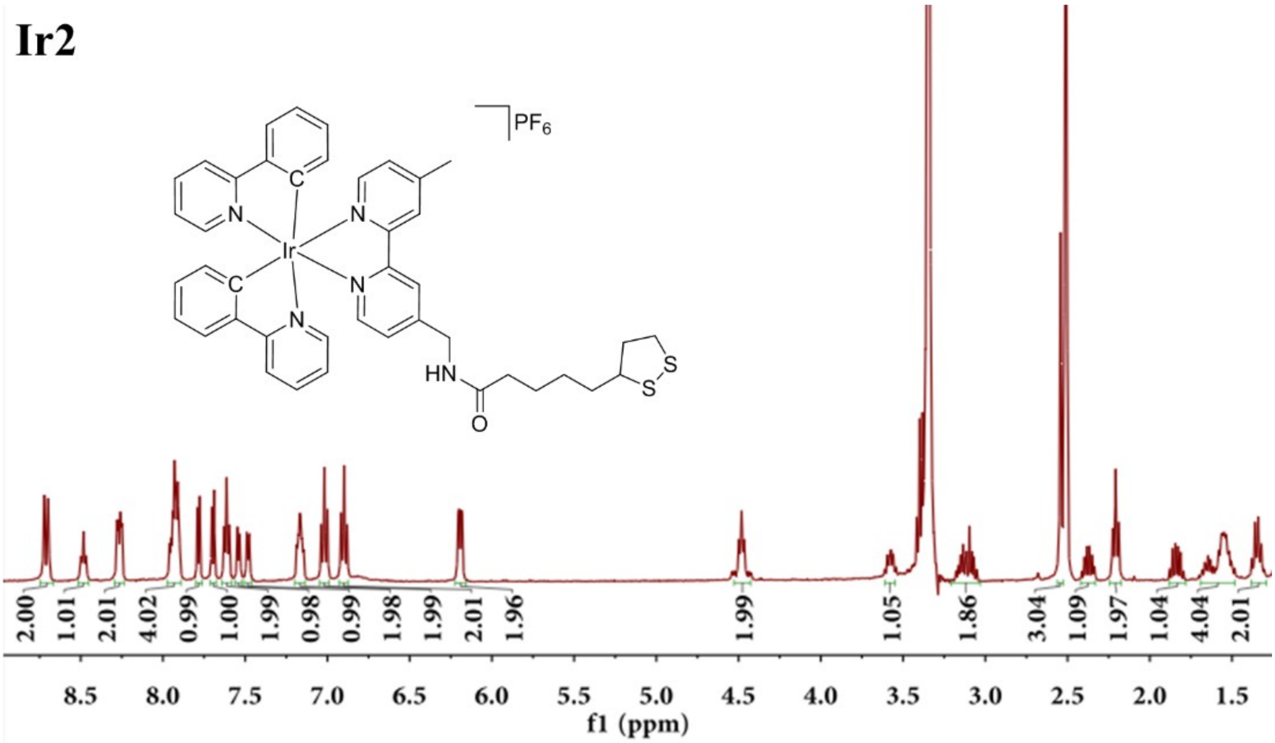

Fig. S7 ^{1}H NMR spectrum (400 MHz, d_6-DMSO) of complex **Ir2**.

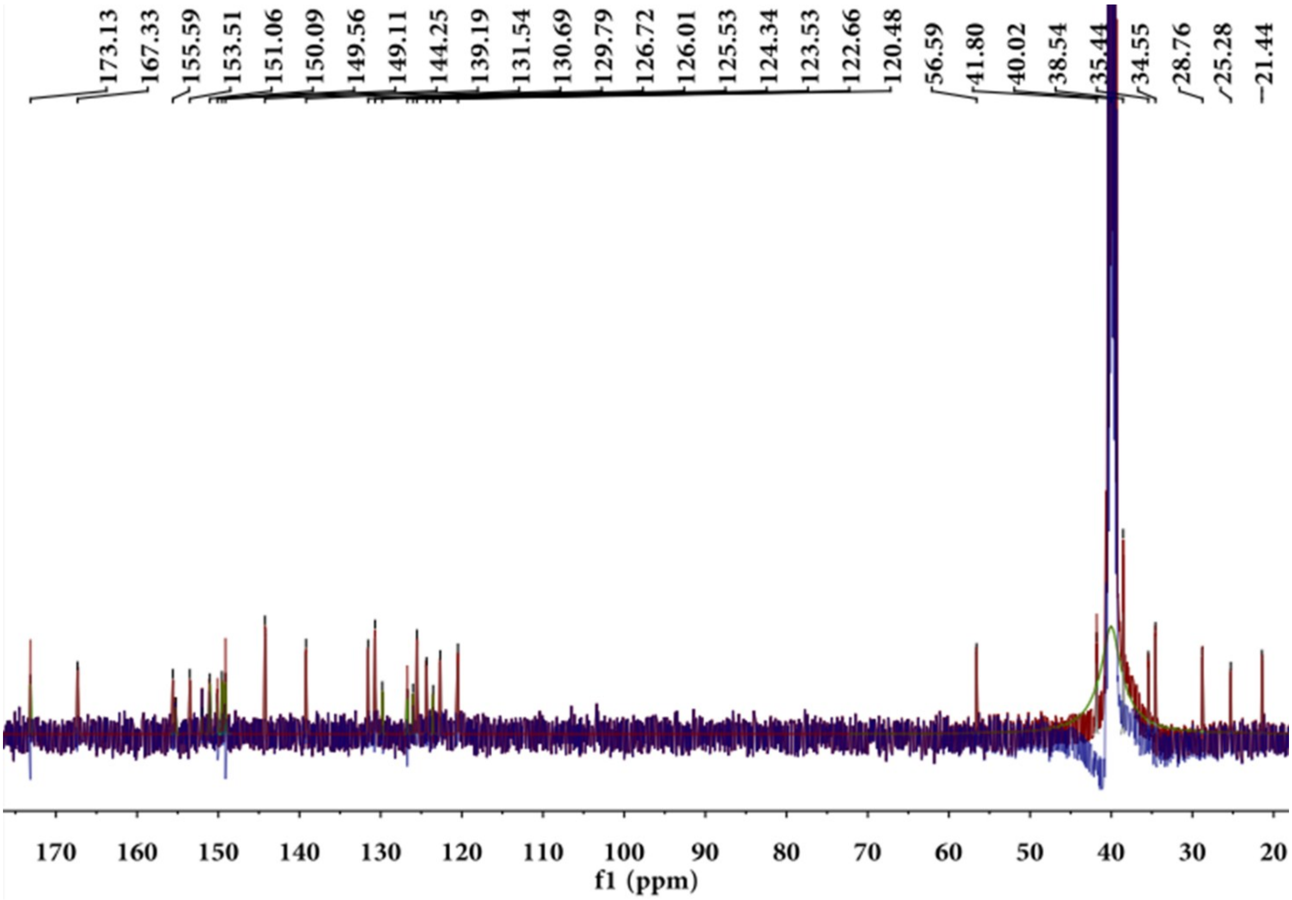

Fig. S8 ^{1}C NMR spectrum (101 MHz, d_6-DMSO) of complex **Ir2**.

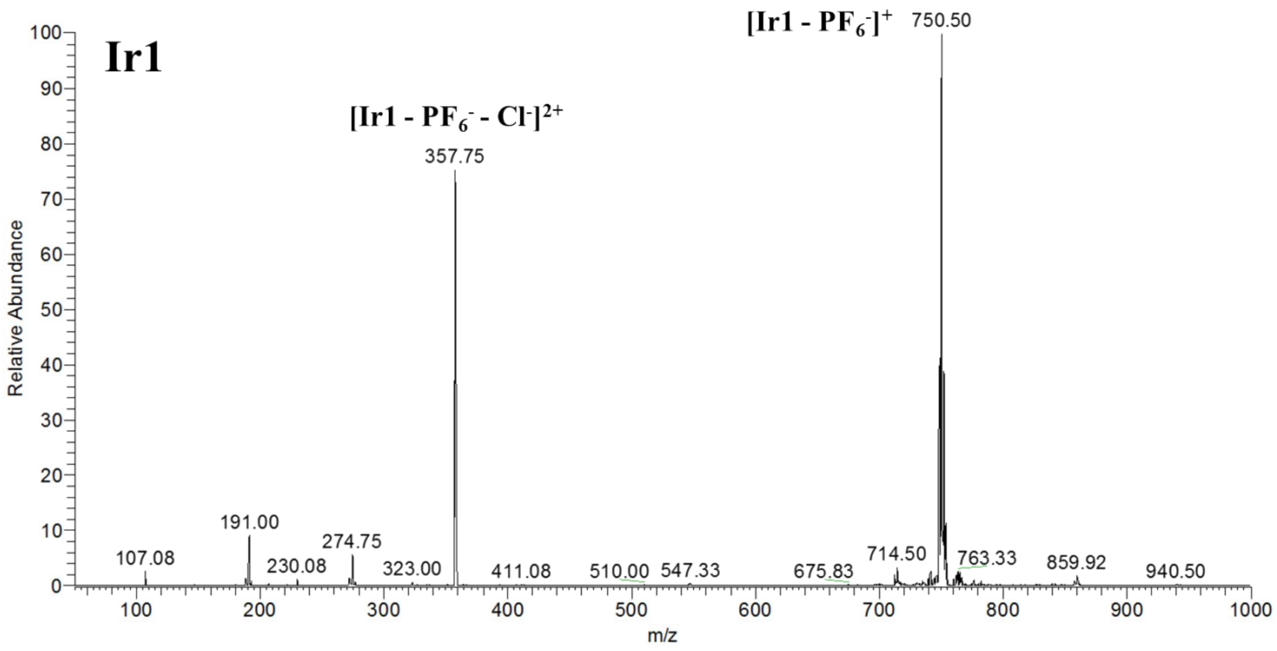

Fig. S9 ESI-MS spectrum (CH_3OH) of complex **Ir1**.

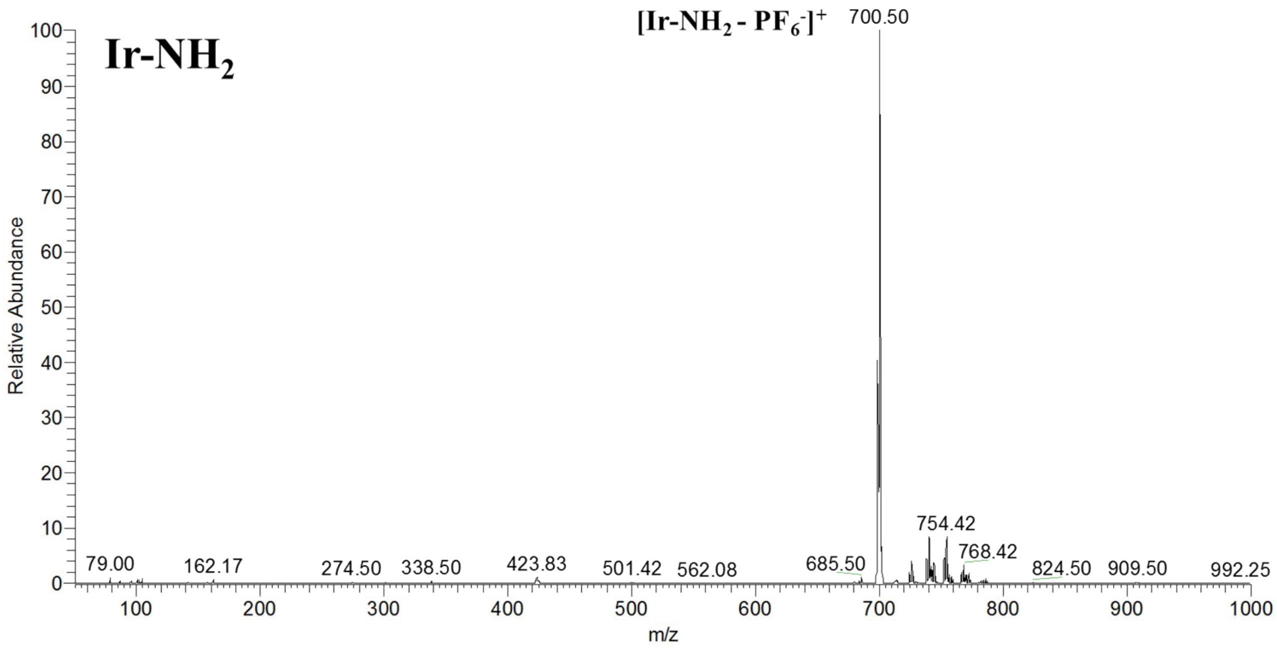

Fig. S10 ESI-MS spectrum (CH_3OH) of complex **Ir-NH$_2$**.

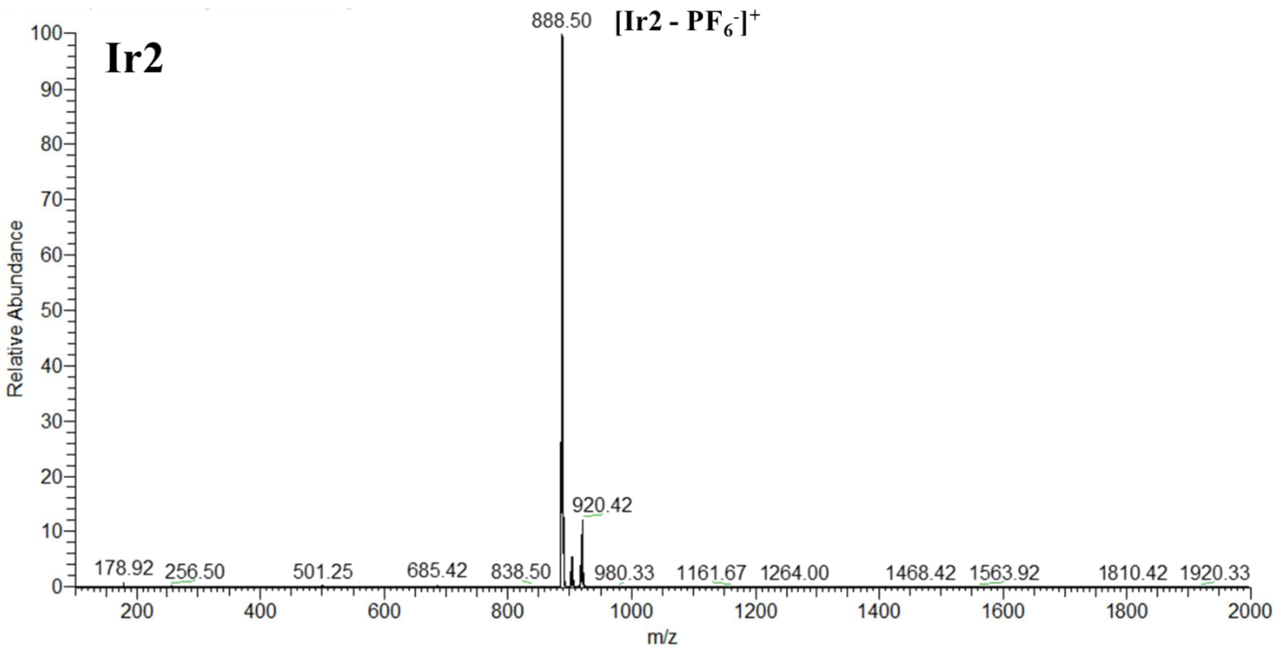

Fig. S11 ESI-MS spectrum (CH_3OH) of complex **Ir2**.

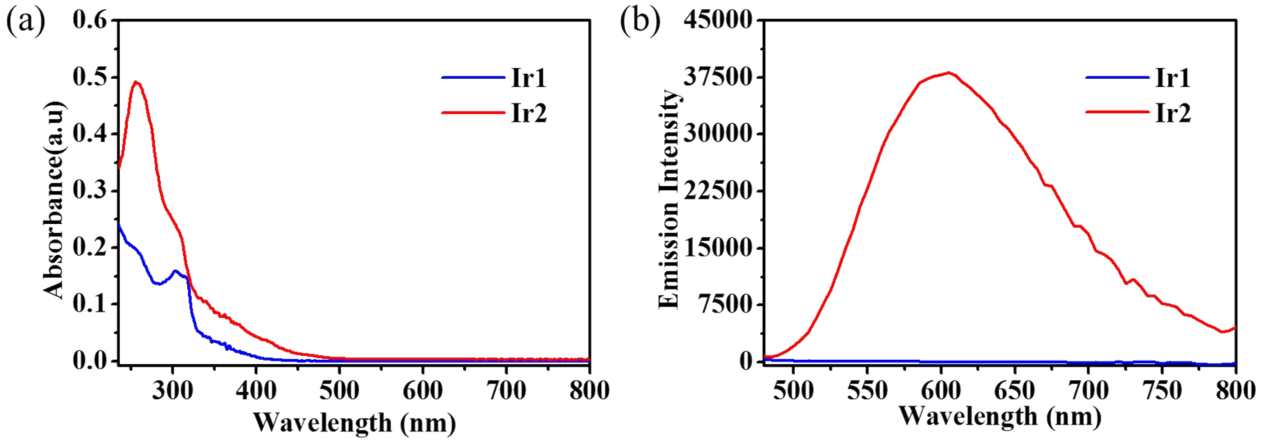

Fig. S12 (a) UV-vis spectra and (b) Emission spectra with the excitation wavelength of 405 nm for 10 μM solution of complexes **Ir1** and **Ir2** in DMSO/H_2O (v/v, 1:99) at 298 K.

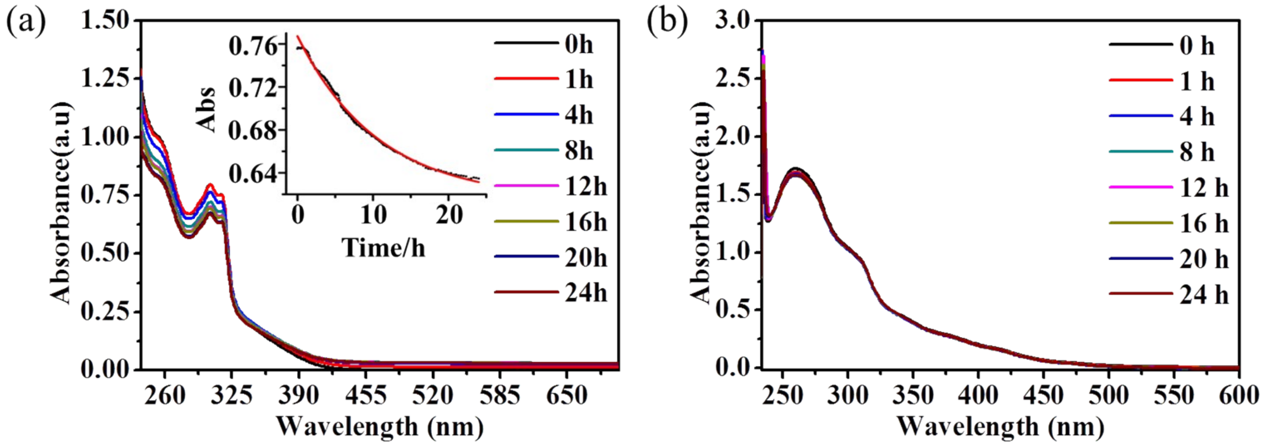

Fig. S13 UV-vis spectra for a 50 μM solution of complexes in DMSO/H_2O (v/v, 5:95) recorded over a period of 24 h at 298 K. (a) **Ir1**; (b) **Ir2**. Inset: Absorbance at 314 nm decreased with time.

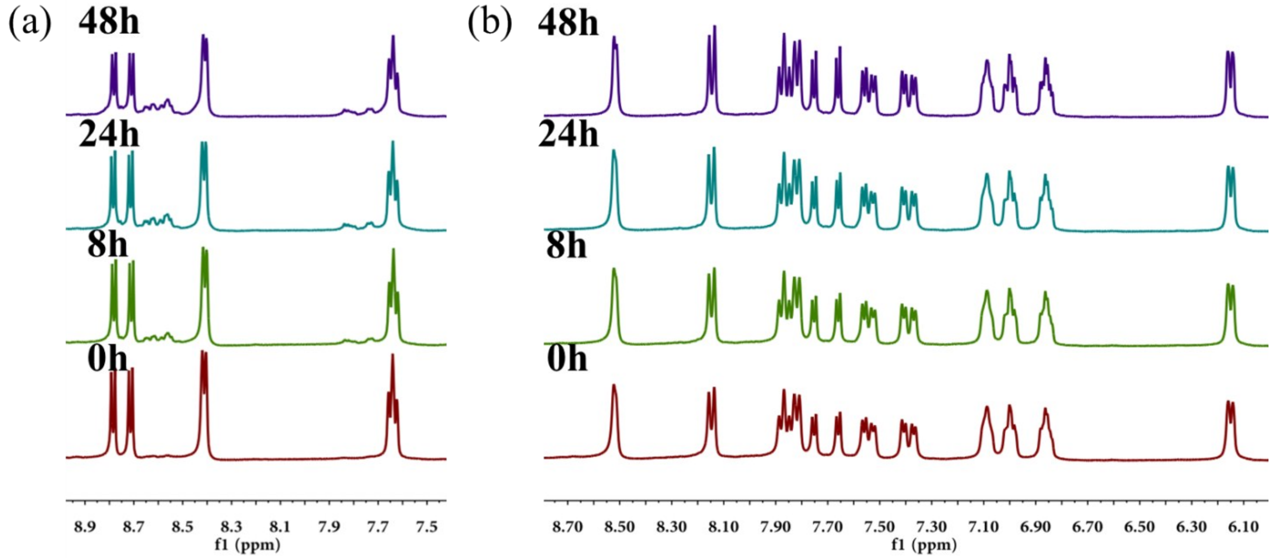

Fig. S14 ^{1}H NMR spectra showing the stability of complexes (10 mM) in d_6-DMSO/D_2O (v/v, 3:1) recorded over a period of 48 h at 298 K. (a) **Ir1**; (b) **Ir2**.

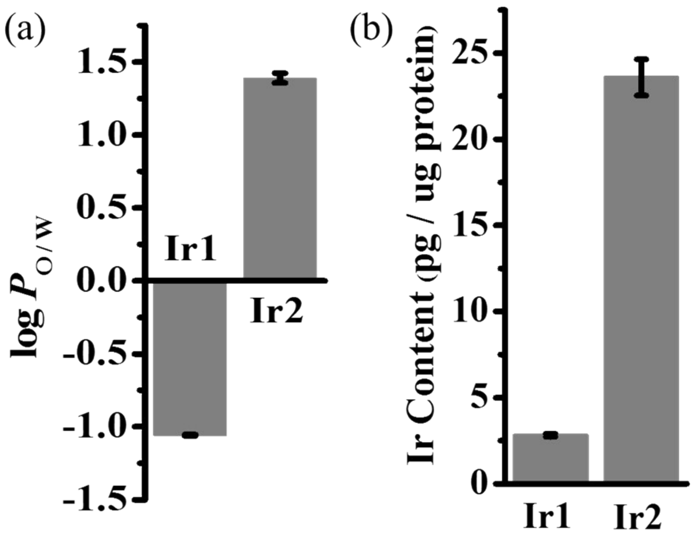

Fig. S15 (a) Log $P_{o/w}$ values of complexes **Ir1** and **Ir2**. (b) Iridium content uptaken by A549 cells pretreated with complexes **Ir1** and **Ir2** (10 μM) for 4 h at 310 K.

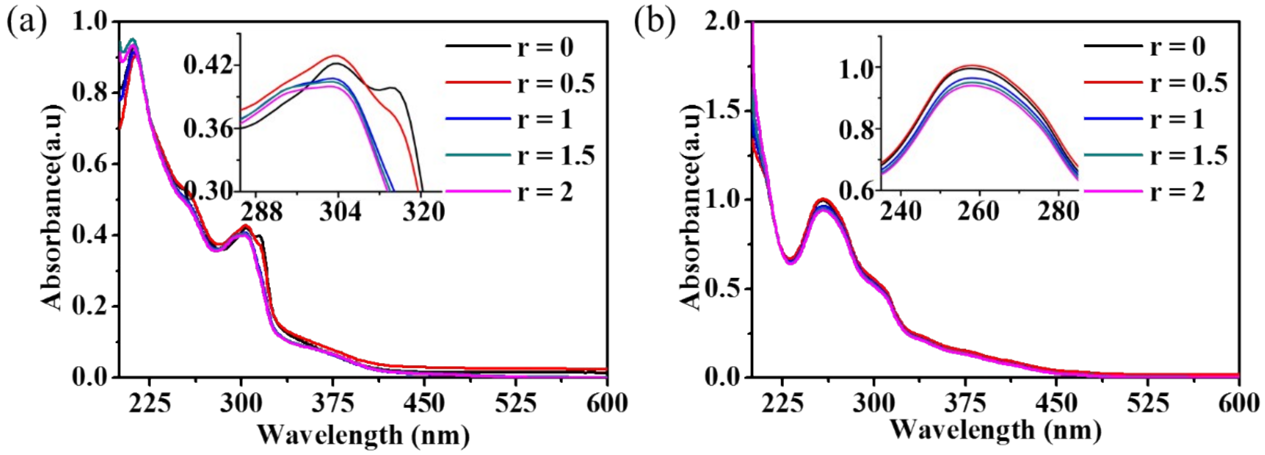

Fig. S16 UV-vis spectrum for different ratio of GSH (r = 0.5, 1, 1.5, 2) and complexes in CH_3OH/H_2O (v/v, 5:95) at 298 K, where the complex concentration was set at 30 μM. (a) **Ir1**; (b) **Ir2**. Inset: Magnification of the spectrum for **Ir1** and **Ir2**, respectively.

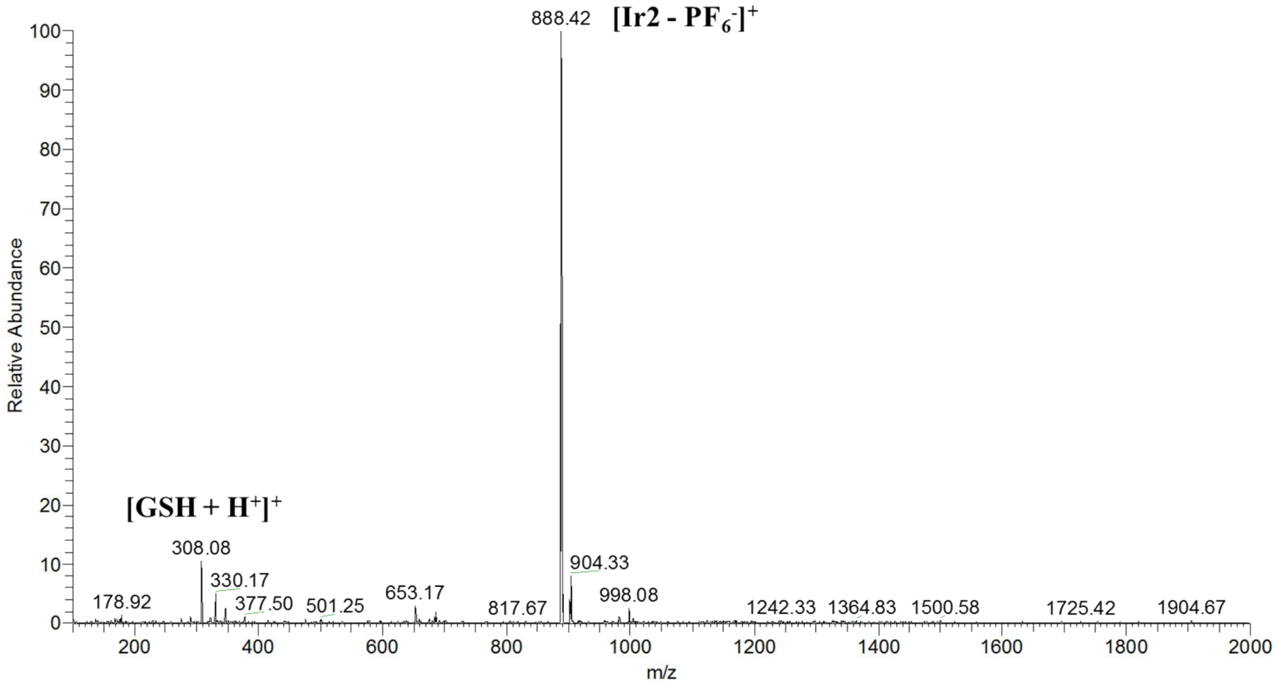

Fig. S17 ESI-MS spectrum for reaction between **Ir2** (1 mM) and GSH (10 mM) in CH_3OH/H_2O (v/v, 1:1) after incubation for 6 h at 310 K.

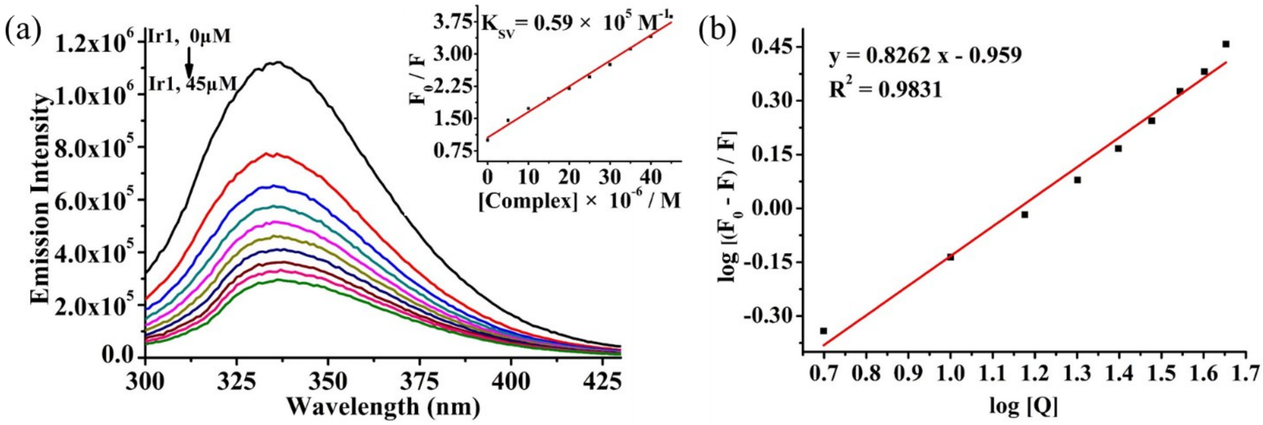

Fig. S18 (a) Emission spectra of BSA quenched by complex **Ir1**. BSA (5 μM) was incubated with **Ir1** (0, 5, 10, 15, 20, 25, 30, 35, 40, 45 μM) at 37 °C for 5 min. λ_{ex} = 285 nm. Inset: Stern-Volmer plot of F_0/F against the concentration of complex **Ir1**. (b) Plot of log[(F_0-F)/F] vs. log[Q] for the interaction of BSA with complex **Ir1**.

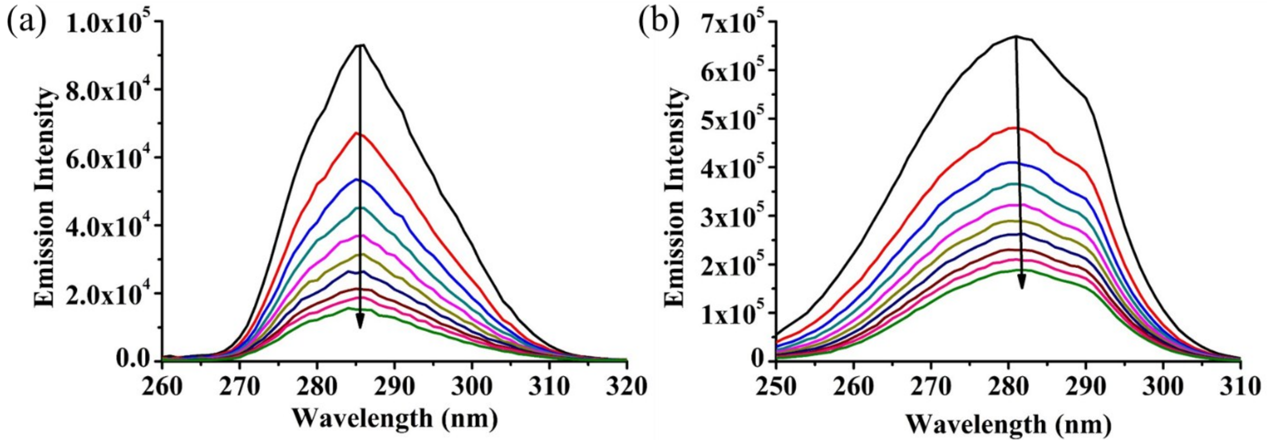

Fig. S19 Synchronous spectra of BSA (5 μM) in the presence of increasing amounts of complex **Ir1** (0-45 μM) with a wavelength difference of Δλ = 15 nm (a) and Δλ = 60 nm (b).

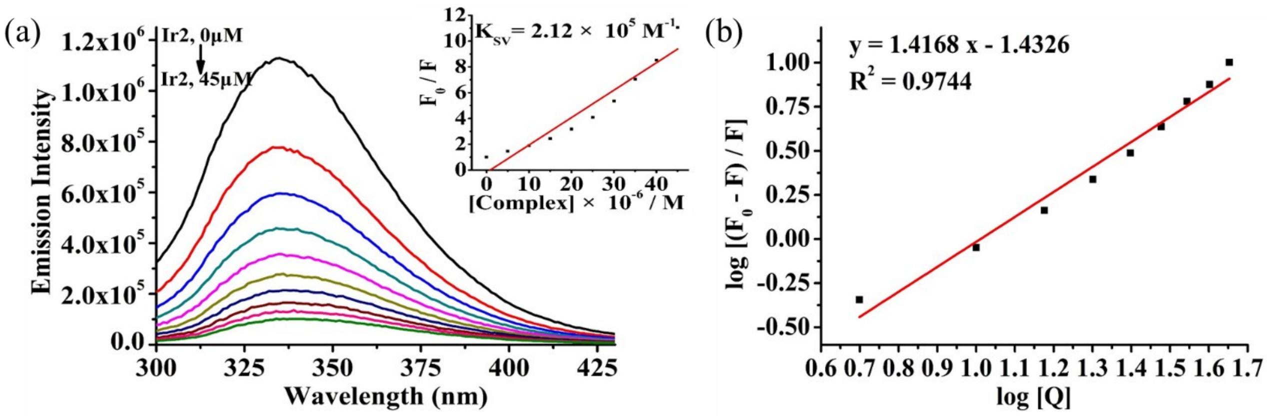

Fig. S20 (a) Emission spectra of BSA quenched by complex **Ir2**. BSA (5 μM) was incubated with **Ir2** (0, 5, 10, 15, 20, 25, 30, 35, 40, 45 μM) at 37 °C for 5 min. λ_{ex} = 285 nm. Inset: Stern-Volmer plot of F_0/F against the concentration of complex **Ir2**. (b) Plot of log[(F_0-F)/F] vs. log[Q] for the interaction of BSA with complex **Ir2**.

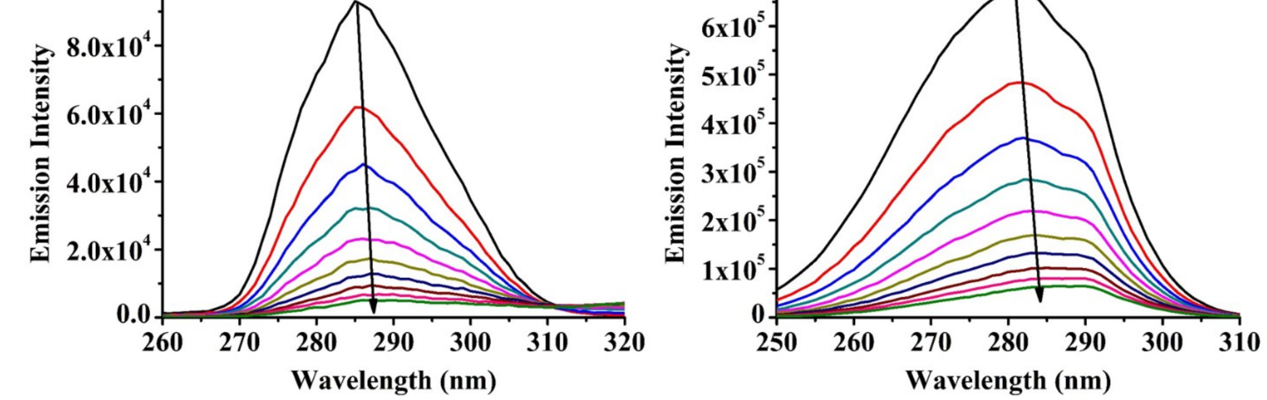

Fig. S21 Synchronous spectra of BSA (5 μM) in the presence of increasing amounts of complex **Ir2** (0-45 μM) with a wavelength difference of (a) $\Delta\lambda$ = 15 nm and (b) $\Delta\lambda$ = 60 nm.

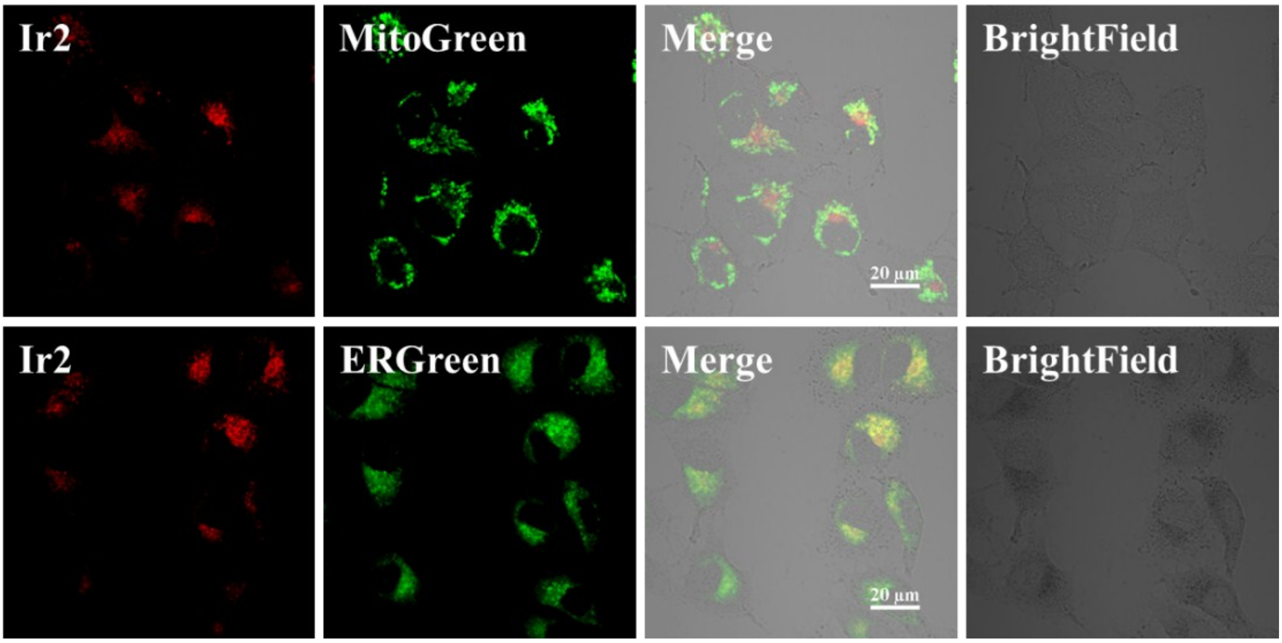

Fig. S22 Intracellular colocalization of **Ir2** with MitoGreen and ERGreen probes observed by confocal microscopy. A549 cells were incubated with **Ir2** (10 μM, 4 h) and then stained with MitoGreen (200 nM, 30 min) and ERGreen (2 μM, 30min). **Ir2**: λ_{ex} = 405 nm; λ_{em} = 600 ± 20 nm. MitoGreen: λ_{ex} = 488 nm; λ_{em} = 516 ± 20 nm. ERGreen: λ_{ex} = 488 nm; λ_{em} =520 ± 20 nm. Scale bar: 20 μm.

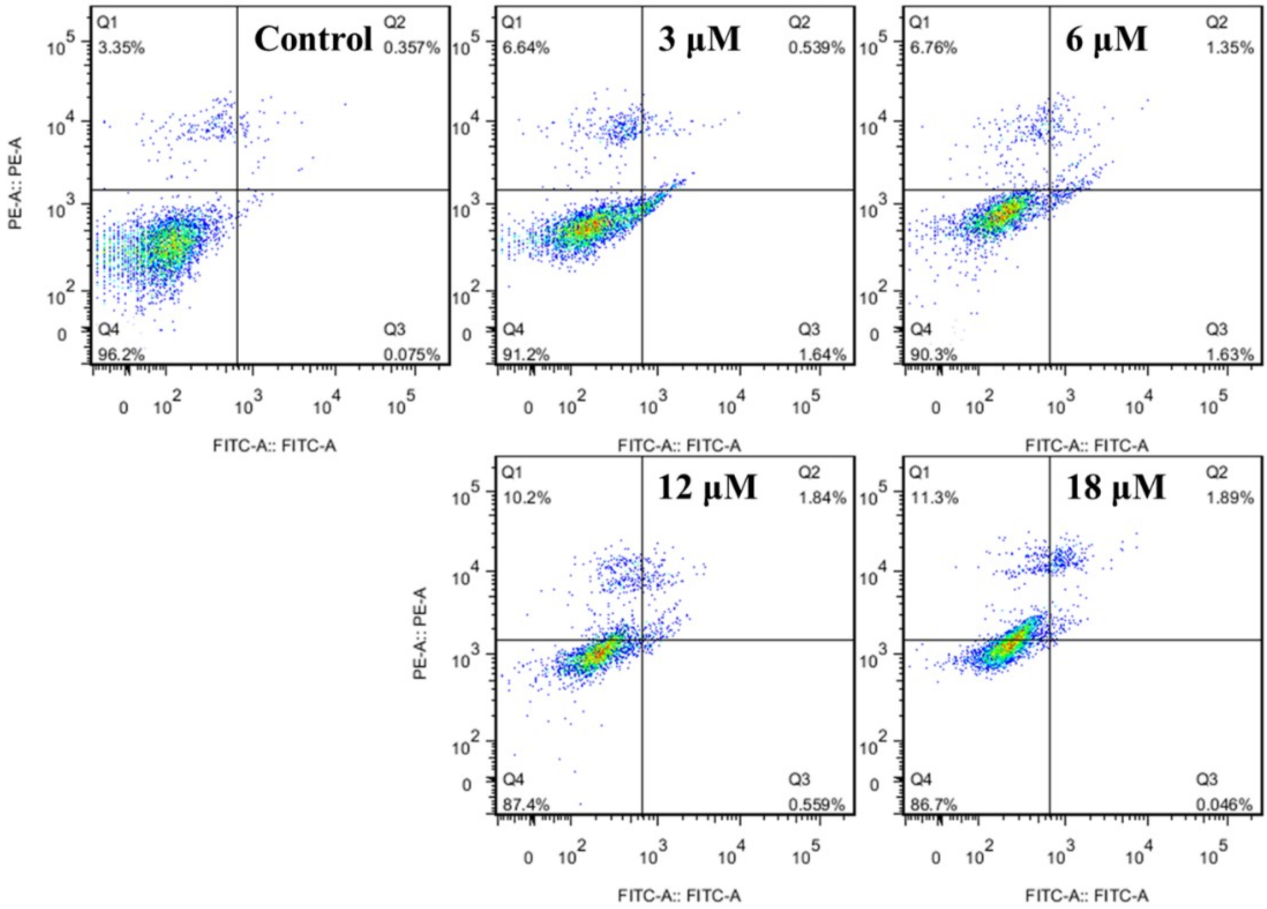

Fig. S23 Apoptosis analysis of A549 cells after exposure to the indicated concentrations of **Ir2** for 24 h at 310 K determined by annexin V and PI double staining measured by flow cytometry.

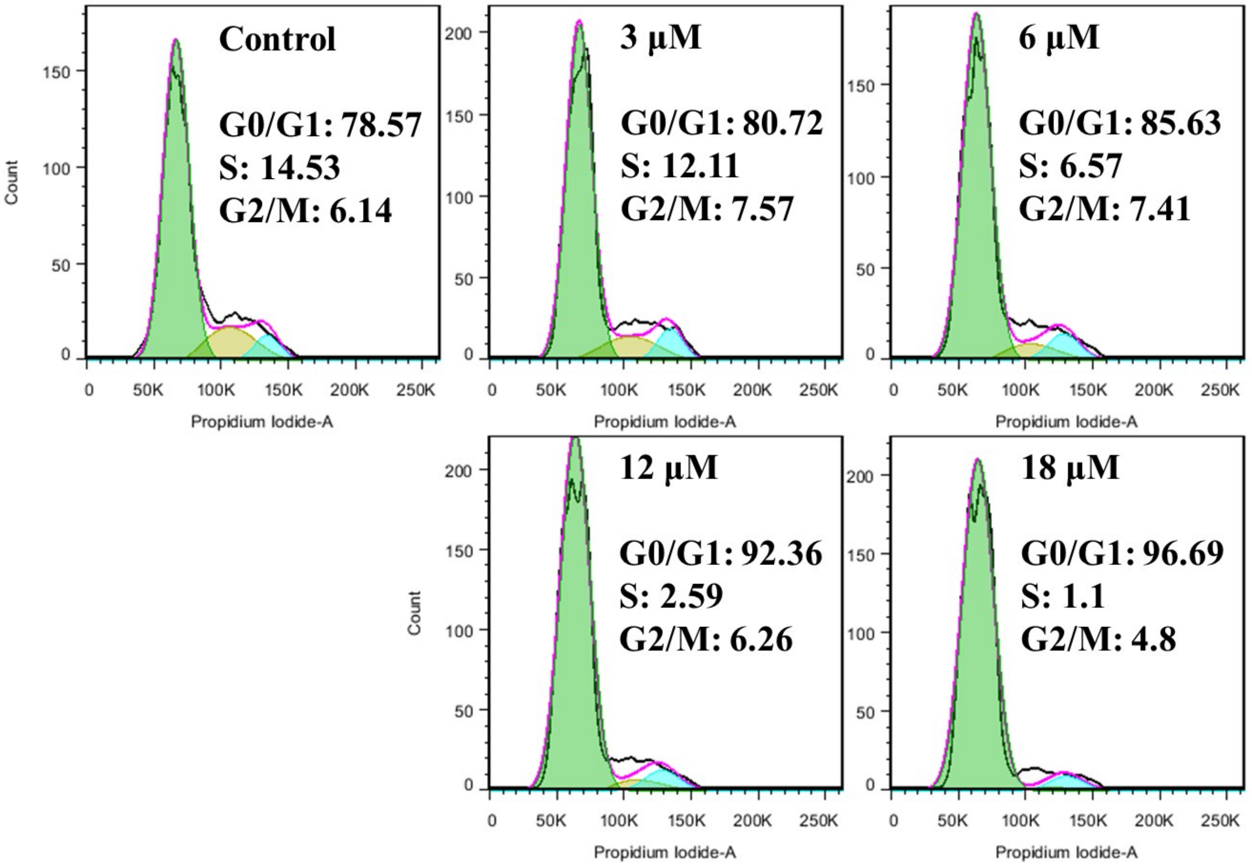

Fig. S24 Cell cycle analysis of A549 cancer cells after exposure to **Ir2** at different concentrations for 24 h at 310 K. Cell staining for flow cytometry was carried out using PI.

Supplementary references

1 C. Busche, P. Comba, A. Mayboroda and H. Wadepohl, *Eur. J. Inorg. Chem.*, 2010, 1295-1302.

2 Z. Fang, A. Ito, S. Keinan, Z. Chen, Z. Watson, J. Rochette, Y. Kanai, D. Taylor, K. S. Schanze and T. J. Meyer, *Inorg. Chem.*, 2013, **52**, 8511-8520.

3 J. J. Cao, C. P. Tan, M. H. Chen, N. Wu, D. Y. Yao, X. G. Liu, L. N. Ji and Z. W. Mao, *Chem. Sci.*, 2017, **8**, 631-640.

4 C. Wang, J. Liu, Z. Tian, M. Tian, L. Tian, W. Zhao and Z. Liu, *Dalton. Trans.*, 2017, **46**, 6870-6883.

5 Z. Tian, J. Li, S. Zhang, Z. Xu, Y. Yang, D. Kong, H. Zhang, X. Ge, J. Zhang and Z. Liu, *Inorg. Chem.*, 2018, **57**, 10498-10502.

6 M. E. Pacheco and L. Bruzzone, *J. Lumin.*, 2013, **137**, 138-142.

7 C. R. Wade and M. Dincă, *Dalton. Trans.*, 2012, **41**, 7931-7938.

8 Z. Cheng, *J. Lumin.*, 2012, **132**, 2719-2729.

9 R. Cao, J. Jia, X. Ma, M. Zhou and H. Fei, *J. Med. Chem.*, 2013, **56**, 3636-3644.
